# Phylogeny and structural modeling of the transcription factor CsqR (YihW) from *Escherichia coli*

**DOI:** 10.1038/s41598-024-58492-y

**Published:** 2024-04-03

**Authors:** Anna A. Rybina, Roman A. Glushak, Tatiana A. Bessonova, Artemiy I. Dakhnovets, Alexander Yu. Rudenko, Ratislav M. Ozhiganov, Anna D. Kaznadzey, Maria N. Tutukina, Mikhail S. Gelfand

**Affiliations:** 1 Skolkovo Institute of Science and Technology, Moscow, Russia 121205; 2 Faculty of Biology, Lomonosov Moscow State University, Moscow, Russia 119234; 3 Institute of Cell Biophysics RAS (Federal Research Center “Pushchino Scientific Center for Biological Research RAS”), Pushchino, Russia 142290; 4 Belozersky Institute of Physico-Chemical Biology, Lomonosov Moscow State University, Moscow, Russia 119991; 5 Institute for Information Transmission Problems RAS, Moscow, Russia 127051

**Keywords:** Computational biology and bioinformatics, Microbiology, Molecular biology

## Abstract

CsqR (YihW) is a local transcription factor that controls expression of *yih* genes involved in degradation of sulfoquinovose in *Escherichia coli*. We recently showed that expression of the respective gene cassette might be regulated by lactose. Here, we explore the phylogenetic and functional traits of CsqR. Phylogenetic analysis revealed that CsqR had a conserved Met25. Western blot demonstrated that CsqR was synthesized in the bacterial cell as two protein forms, 28.5 (CsqR-l) and 26 kDa (CsqR-s), the latter corresponding to start of translation at Met25. CsqR-s was dramatically activated during growth with sulfoquinovose as a sole carbon source, and displaced CsqR-l in the stationary phase during growth on rich medium. Molecular dynamic simulations revealed two possible states of the CsqR-s structure, with the interdomain linker being represented by either a disordered loop or an ɑ-helix. This helix allowed the hinge-like motion of the N-terminal domain resulting in a switch of CsqR-s between two conformational states, “open” and “compact”. We then modeled the interaction of both CsqR forms with putative effectors sulfoquinovose, sulforhamnose, sulfoquinovosyl glycerol, and lactose, and revealed that they all preferred the same pocket in CsqR-l, while in CsqR-s there were two possible options dependent on the linker structure.

## Introduction

Transcription factors (TFs), also referred to as regulators, are essential for bacterial survival and adaptation to changing environments. TFs regulate expression of genes through multiple mechanisms^1^. The activity of regulators is often modulated by the presence of small molecule effectors^2,3^. These effectors bind to the TF and alter its conformation, resulting in changes in its DNA-binding affinity and/or oligomerization^3^. This allows the regulator to respond to environmental changes and adjust gene expression accordingly. Some TFs may interact with several effectors, often intermediates of the metabolic pathway that is under control of the respective TF. For instance, CggR of *Bacillus subtili*s from the SorC/DeoR family, regulating genes of glycolysis, has several ligands that are metabolites of glucose conversion, in particular, fructose-1,6-bisphosphate, dihydroxyacetone phosphate, glucose-6-phosphate, and fructose-6-phosphate^4^. AraC of *Escherichia coli* from the AraC family, regulating L-arabinose transport and metabolism, has effectors L-arabinose^5^ and D-fucose^6^. Other examples of regulators with several effectors are MarR (aromatic acids and antibiotics)^7^, PlaR (D-galacturonate and L-ascorbate)^8^, RutR (uracil and thymine)^9^, and AlsR (D-allose, D-ribose)^10^.

Genes encoding TFs are often co-localized with the TF’s targets^3,4^. The object of this study, local regulator CsqR (formerly YihW) from *Escherichia coli*, is one of such regulators. CsqR is encoded by the *csqR* (*yihW)* gene located within the *yih* cassette. The *yih* cassette consists of ten genes^11,12^, at least seven of which, *yihO* (transporter), *yihQ* (sulfoquinovosidase), *yihR* (mutarotase), *yihS* (isomerase), *yihV* (kinase), *yihU* (reductase), and *yihT* (aldolase), are responsible for the degradation of sulfoquinovose (SQ), a sulfonated derivative of glucose, via the sulfo-Embden–Meyerhof–Parnas (sulfo-EMP) pathway^11,13–15^. We had suggested a repressor role of CsqR for these genes^12^, which was further confirmed by Shimada et al.^15^.

CsqR belongs to the DeoR family^16^ comprising regulators with an N-terminal DNA-binding domain with the helix-turn-helix (HTH) motif and a C-terminal effector-sensing domain^16,17^. SQ was shown to act as an effector of CsqR^15^. It has been also reported that both sulfoquinovosyl glycerol (SQG)^15^, a glycoside of SQ, and sulforhamnose (SR)^18^, an intermediate of the SQ catabolism, might decrease CsqR binding to DNA. SR and sulfofructose (SF) are produced from β-SQ by the YihS isomerase^18^.

The oligomeric state of CsqR (YihW) is not well studied yet. In^15^, AFM showed that CsqR mainly existed as a monomer in the absence of DNA, but formed large aggregates when mixed with the *yihUV* probe, indicating it is a member of TFs that exhibit high cooperativity once bound to target DNA. Other DeoR-type transcription factors tend to be in oligomeric state as well: UlaR from *E. coli* is tetrameric in a DNA-bound state, and homodimeric in a DNA-free state^19^, DeoR aggregates into an octamer^20^, and AgaR was reported to form tetramers^21^. Based on these observations, we may speculate that CsqR (YihW) might function as an oligomer, at least as a homodimer, in a DNA-bound state.

At the same time, a comparative genomics analysis demonstrated similarity in the functional composition of the *yih* cassette, and a cassette of bacteria from class Bacilli responsible for the lactose catabolism^12^. Further, activation of several *yih* genes, namely *yihV* (kinase), *yihT* (aldolase), and *yihS* (isomerase), during growth with lactose was confirmed. Hence, we assumed that the *yih* cassette may also be regulated by lactose. In addition, expression of the *csqR* gene itself was up-regulated by lactose during the exponential phase of growth and repressed under depletion of this carbon source hinting at a dual role of CsqR as a regulator for the *yih* genes. Indeed, CsqR acted as a repressor for *yihV*, *yihT,* and *yihS* during growth with glucose, and had no effect during growth on lactose^12^. Recently, a microarray study on *E. coli* also showed activation of the *csqR* gene in response to lactose during glucose-lactose diauxic shift at the growth arrest phase^22,23^. These observations suggest that lactose might serve, together with SQ, as an effector molecule for CsqR. However, gel shift did not corroborate the effector role of lactose for CsqR^15^.

The *yih* genes were also identified in Actinobacteria^24^ although the *csqR* (*yihW*) ortholog was not observed. The *yih* genes might be co-localized with genes from another sulfoglycolytic cluster known as sulfo-EMP2 that also degrades SQ via the sulfo-EMP pathway^25,26^. The sulfo-EMP2 locus contains genes that are non-orthologous to the *yih* genes but share similar functions, namely, *sqvD* (isomerase), *sqiA* (aldolase), *slaB* (reductase), and a putative *sqvB* (mutarotase)^25^. The *yih* locus in *Arthrobacter* sp. strain AK01 was reported to include the *sqgA* gene that codes for sulfoquinovosidase (SQase) as an alternative to YihQ^27^. However, the co-localization patterns of the *csqR* gene have not been fully described, and the distribution of its homologs among bacterial species remains unclear.

Hence, an uncertainty still exists about the CsqR functions. Here we aimed to study the evolution of CsqR, its synthesis in *E. coli* during growth on different carbon sources, and possible patterns of the CsqR interaction with candidate effectors.

## Methods

### Strains and plasmids

All strains and plasmids used in this work are listed in Table 1.

**Table 1:** Strains and plasmids used in the study.

Strain	Description	References
K-12 MG1655	Wild type F- lambda- ilvG- rfb-50 rph-1	^28^
K-12 MG1655_yihW_his	K-12 MG1655 with 6xhis-tag added to the 3’-end of the *csqR (yihW)* gene	This work
BL21*(DE3)	F- ompT hsdSB (rB-mB-) gal dcm rne131 (DE3)	^29^
BL21-CodonPlus(DE3)-RIL	F- ompT hsdSB (rB- mB-) gal dcm (DE3) *endA* Hte [*argU proL*Camr] [*argU ileY leuW* Strep/Spec^R^ ]	^30^
OverExpress C41(DE3)	F- ompT hsdSB (rB- mB-) gal dcm (DE3)	^31^
Plasmids		
pGEM ∆Xba	Expression vector based on pGEMEX1 (Promega) Amp^R^	^32^
pGEMEX_yihW_TEV_his	Vector overproducing C-6x-his-tagged CsqR (YihW) protein, Amp^R^	This work

### Tools and databases

Complete bacterial genomes and translated protein-coding DNA sequences (CDS) were taken from the NCBI RefSeq database^33^. The Pfam protein database version 35.0 (Pfamseq) was retrieved from the EMBL-EBI server^34^. Metadata for cross-referencing protein unique identifiers (ID) was obtained from UniProt^35^.

Homologs of the CsqR protein from *E. coli* str. K-12 MG1655 (NC_000913.3) were found in Pfamseq using phmmer v3.1b2 with the default parameters^36^. The respective protein sequences were obtained with esl-sfetch vh3.1b2^37^ and used as a local database for additional protein BLAST search with CsqR from *E. coli* str. K-12 MG1655 as a query. Records with E-value < 0.001 were selected. UniProt Knowledgebase accession numbers of proteins derived from the Pfamseq were cross-referenced to GenBank protein ID using protein ID mapping metadata from UniProt, and bacterial CsqR homologs were retained. To obtain all possible close CsqR homologs, we also identified genome fragments homologous to the *csqR* gene. First, genome regions homologous to the *csqR* gene of *E. coli* str. K-12 MG1655 were identified using the NsimScan tool v1.1.84^38^ against bacterial genomes (command line arguments: −v −k 8 −t 150 –it 55 –xt 55 –mdom). For each homologous region, midpoint genomic location was calculated and used to obtain respective protein sequences from translated CDS records. CsqR homologs obtained by both approaches were pooled into a single local database for the BLASTP analysis.

To check if genes of the *yih* cassette and sulfo-EMP2 locus are co-localized with *csqR* in other bacterial species, we used NsimScan^38^ to identify genomic regions homologous to the *yih* genes of *E. coli* str. K-12 MG1655, the *sqgA* gene of *Arthrobacter* sp. strain AK01^27^, and the sulfo-EMP2 genes of *Alkalicoccus urumqiensis* BZ-SZ-XJ18^25^. To validate homology between gene products, we compared protein sequences using the Needleman-Wunsch algorithm with default settings^39^. The respective genomic loci were visualized with the gggenes R package^40^.

### Construction of phylogenetic trees

Protein sequences were aligned with MAFFT v7.475 with the default parameters^41^. The phylogenetic tree was constructed using FastTree v2.1.11 No SSE3 with the default options^42^. To reduce the number of nodes in the phylogenetic tree, protein sequences from organisms of the RefSeq category “representative” or “reference genome” were selected. Then, the representative protein sequences were obtained based on linear clustering via MMseqs2 v13-45111^43^. To prune the phylogenetic tree and the respective alignment for tree visualization, Biopython v1.76^44^ was used. Taxonomy of organisms whose protein sequences were present in the tree, was obtained via TaxonKit v0.8.0^45^. The tree was displayed using the ggtree R package^46^. Multiple sequence alignment was plotted with the ggmsa R package^47^.

### Molecular dynamic simulations and docking

Three-dimensional (3D) structures of SQ, SR, SQG, glucose, and lactose were retrieved from the PubChem database^48^ (PubChem CID 86289062, 162640041, 100920818, 5793, and 6134, respectively). For docking, hydrogens were added and geometry of each molecule was optimized with Avogadro v1.2.0^49^. Then, non-polar hydrogens were merged, and Gasteiger charges were assigned to both molecules using AutoDockTools4 v1.5.6^50^.

The three-dimensional structure of CsqR (RefSeq accession: NC_000913.3) was predicted using I-TASSER^51^ and AlphaFold v2.1.0^52^ with the default options. To estimate the model confidence, AlphaFold per-residue confidence score (pLDDT)^53^ was obtained from the AlphaFold output and plotted across the protein length.

AlphaFold and I-TASSER models were used as starting coordinates in 2 µs molecular dynamic simulations (MDS) using GROMACS version 2023.2^54^. Preliminary input files were generated by the CHARMM-GUI Input Generator (Solution Builder)^55^, with most parameters being set as default except for the following: (1) Na^+^ and Cl^−^ were used as basic ion types to neutralize the system; (2) Amber ff19SB^56^ was chosen as force field; (3) temperature coupling using velocity rescaling with a stochastic term was specified (tcoupl = V-rescale). To check behavior of protein regions with ambiguous secondary structure, an additional run of 1 µs simulation was performed with increased conformational mobility of protein by setting its temperature in the system to 350 K.

After simulations, the trajectory corrected for periodicity was extracted (gmx trjconv -pbc mol -center), the respective frames were converted to the gro file format (gmx trjconv) and inspected in PyMol v2.5.2^57^. The secondary structure elements were assigned using the DSSP algorithm^58^ via the gmx dssp module. To get representative structural conformations, the corrected trajectory was clustered (gmx cluster -method gromos -cutoff 0.6). Cluster membership of each frame was plotted across the simulation time using a custom Python script. Centroids of the most populated clusters presented closer to the end of simulation were selected for subsequent analysis. Then, representative structural conformations underwent energy minimization with the same parameters used earlier as a part of MDS.

Contact Map Explorer, a module of the MDTraj Python package^59^, was used to examine the frequency of residue-residue contacts in MDS trajectories with default parameters. To identify potential interatomic interactions important for stabilizing one protein conformation compared to another, the difference of frequencies 0.6 was used as a threshold.

Flexible structural alignment was performed using FATCAT^60^ with the default parameters. Structural superposition was done via the align command in PyMol v2.5.2^57^. Structure similarity search against the Protein Data Bank was performed using Dali^61^.

Docking was done using AutoDock Vina v1.2.3-52-g92d1779^62^. Docking output was visualized in PyMol v2.5.2^57^. Binding modes were assessed based on the estimated binding free energy and the Root Mean Square Deviation (RMSD) values calculated relative to the reference mode. The electrostatic potential surface of proteins was computed using the Adaptive Poisson-Boltzmann Solver (APBS) program^63^. Binding pockets were obtained via Fpocket v4.0^64^ with default parameters.

### Production of the CsqR protein

CsqR was cloned into the pGEM ∆Xba plasmid^65^ together with its own Shine-Dalgarno box to minimize the possible toxic effect of uncontrollable production of the transcription factor (primers: 5’-AATGTCTAGATGATGGTTTTTCGA-3’ (*yihW_F*) and 5’-TGTGTCTAGATATGAAGCCAGTCAGTGATGGTGATGGTGATGGCCCTGAAAATACAGGTTTTCCGCGTCTTCCTGG-3’ (*yihW_R_TEV*)). The resulting plasmid was chemically transformed into three different BL strains (Table 1). In addition to typical BL21*(DE3), its derivative C41 optimized for production of toxic proteins was used, as well as BL21-CodonPlus(DE3)-RIL possessing additional copies of the *argU*, *ileY*, and *leuW* genes encoding rare tRNAs for AGA/AGG, AUA, and CUA codons, respectively. Overnight cultures of transformants were grown aerobically at 37 °C in the standard Luria–Bertani (LB) medium with 100 ug/ml ampicillin for 16 h and then transferred 1:200 to 50 ml flasks containing 20 ml of the same medium. Cells were grown till exponential phase (OD_600_ = 0.2–0.3), and the synthesis of recombinant CsqR was induced with IPTG (final concentrations 0.02, 0.05, and 0.1 mM). Samples were taken after 5 and 16 h of induction. Then OD_600_ was measured using the UV–Vis spectrometer (Thermo, USA), cells from 1 ml of culture were spun down at 10 000 rpm (MiniSpin) (RT), and resuspended in the appropriate volume of BugBuster protein extraction reagent (Novagen, USA; V_BB_ = (OD_600_ V_ml_)*0.015). Further separation of the soluble and insoluble protein fractions was done using the manufacturer’s protocol. Samples were finally dissolved in the 4X loading buffer (0.2 M Tris–HCl (pH 6.8), 0.4 M β-mercaptoethanol, 4% SDS, 0.01% bromophenol blue and 40% glycerol), using the same volume as for BugBuster.

### Sulfoquinovose synthesis

**Figure 1:**
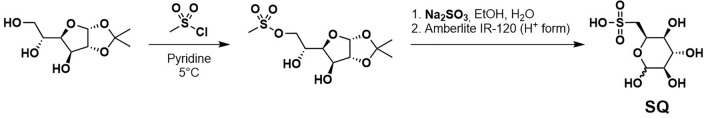
Scheme for the synthesis of Sulfoquinovose (SQ).

SQ synthesis was performed using part of the protocol described previously^66,67^ (Fig. 1).

In a 500 ml round bottom flask equipped with a magnetic stirrer, 7.94 g (1 eq) of 1,2-O-isopropylidene-D-glucofuranose was put, followed by addition of 200 ml of dry pyridine. The mixture was stirred until complete dissolution, and then the flask was placed on an ice bath for 30 min to lower the temperature to 5 °C. Subsequently, 3.07 ml (1.1 eq) of mesyl chloride was added dropwise with vigorous stirring. Addition of the first drop caused the contents of the flask to turn bright yellow. After all portions of mesyl chloride had been added, the solution was allowed to warm to room temperature and left for 4 h. To quench any remaining mesyl chloride, 4 ml of methanol was added. The solvent was then removed under vacuum, resulting in a sticky residue which was dissolved in 100 ml of water. The aqueous layer was extracted four times with 70 ml of ethyl acetate each time. The organic phase was dried using Na_2_SO_4_ and the solvent was removed under vacuum to obtain the crude product as a white solid. The mesylate was further purified using gradient column chromatography on silica gel with a methanol/dichloromethane mixture ranging from 1 to 3% methanol. The R_f_ = 0.37 (DCM/MeOH 96/4), and the yield of the purified product was 10.14 g (94%).

^1^H NMR spectra were recorded on a Bruker AVANCE 600 spectrometer (600.13 MHz). Chemical shifts are given in ppm relative to SiMe_4_.

^1^H NMR (600 MHz, CDCl_3_) δ 5.9 (d, J = 3.6 Hz, 1H), 4.6–4.5 (m, 2H), 4.4–4.3 (m, 2H), 4.3–4.2 (m, 1H), 4.1 (dd, J = 7.8, 2.9 Hz, 1H), 3.1 (s, 3H), 3.1 (d, J = 5.5 Hz, 1H), 2.8 (d, J = 4.4 Hz, 1H), 1.5 (s, 3H), 1.3 (s, 3H).

To prepare the sodium salt of 6-sulfo-1,2-O-isopropylidene-D-glucofuranose, a solution of the previously synthesized mesylate (4 g, 1 eq) in 200 ml of ethanol and 7 g (4 eq) of sodium sulfite dissolved in 200 ml of water were combined and refluxed for 24 h. After this period, excess sodium sulfite was removed by filtration. The resulting filtrate was then evaporated until the volume reached 100 ml and passed through 200 ml of acidic Amberlite IR-120 resin to obtain the desired acid while simultaneously deprotecting the isopropylidene group. The majority of the solvent was removed under vacuum. To ensure complete dryness, the residue was cryodesicated (freeze drying) one day. To remove remaining methanesulfonic acid, the solid product was washed with cooled methanol (10 ml, 4 times) and hexane (10 ml, 2 times), followed by desiccation to obtain a slightly off-brown product (1.47 g, 44%).

^1^H NMR (600 MHz, D_2_O) δ 5.20 (d, J = 3.8 Hz, 1H), 4.66 (d, J = 8.0 Hz, 1H), 4.21 (t, J = 9.6 Hz, 1H), 3.78 (t, J = 9.5 Hz, 1H), 3.71 (t, J = 9.5 Hz, 1H), 3.55 (dd, J = 9.7, 3.7 Hz, 1H), 3.48 (t, J = 9.3 Hz, 1H), 3.39 (d, J = 15.0 Hz, 1H), 3.31–3.23 (m, 3H), 3.10–3.03 (m, 3H). ^13^C NMR (151 MHz, D_2_O) δ 95.1, 91.1, 74.7, 73.2, 71.8, 71.8, 71.5, 71.3, 70.5, 66.9, 51.4, 51.4. Spectral data are in accordance with those previously described^68^.

### Western-blot analysis

To determine what CsqR forms were synthesized in *E. coli* K-12 MG1655 cells during growth on different sugars, Gene doctoring^69^ was used to construct the K-12 MG1655_yihW-6xHis-tag strain, where nucleotides encoding six histidines were added to the 3’-end of the original *csqR (yihW)* gene. Cell cultures were grown in the minimal medium M9 supplemented with 5% (v/v) LB and 0.2% (w/v) of a carbon source, D-glucose, D-galactose, D-fructose, D-glucuronic acid, lactose, or sulfoquinovose. Bacterial cultures were grown aerobically at 37 °C till mid-exponential phase. OD_600_ of each sample was measured using UV–VIS Spectrophotometer (Thermo Scientific, USA). Cells were centrifuged at 10,000 rpm (+ 4 °C) for 10 min, and dissolved in an appropriate volume of the loading buffer calculated as described above. After 10 min of boiling at 97 °C, samples were run onto a denaturing 10% polyacrylamide gel in a standard tris–glycine buffer at 20 mA/gel^70^. The Trans-Blot Turbo System was used to transfer samples onto a PVDF membrane following the manufacturer's protocol (Bio-Rad, USA). The PVDF membrane was blocked for one hour with 5% skimmed milk (Oxoid, UK) in the TBS buffer (50 mM Tris, 273 mM NaCl, pH 8.0). Subsequently, the membrane was incubated with a rabbit polyclonal antibody against the 6x-His tag (1:10,000, Cat № PA-19838, Invitrogen, USA) in 5% skimmed milk diluted in the TBS-T buffer (TBS with 1:1000 Tween-20) for two hours. This was followed by one-hour incubation with a secondary Anti-rabbit IgG HRP-linked antibody (1:10,000, Cat No 7074S, Cell Signaling Technology, USA) in 5% skimmed milk in the TBS-T buffer. After staining with the Luminata Forte HRP substrate (Millipore, USA), the membrane was visualized in the iBright750 Imaging system (Thermo Scientific, USA).

### RNA extraction and qRT-PCR

Cells grown in the same conditions as those used for the western-blot were used for RNA extraction. To monitor expression changes with time, samples were taken after 4 and 6 h of growth. RNA was extracted using TRIZol (Thermo Fisher Scientific, USA) according to the manufacturer’s protocol and then treated with DNAse I (New England Biolabs, USA) for 1 h at 37 ℃. Reverse transcription was made using 1 μg of total RNA, gene specific primers, and MMul-V RevertAid reverse transcriptase (Thermo Fisher Scientific, Lithuania) according to the manufacturer’s protocol. The A DT-lite thermocycler (DNA-Technology, Russia) and SYBR Green I as a fluorescent dye (Invitrogen, USA) were used for quantitative PCR (qRT-PCR). Primers 2 (5’-GCGATCAGCATGAGGAGTTG’-3’) and 3 (5-GGTGATGGTTTTTCGAGGAA-3’) were used to detect expression level of the full-sized mRNA (*csqR*-l), while primers 1 (5’-CCGTATTAACGACGCTGGAA-3’) and 4 (5’-TGATGAGCTGGCAAATCTGC-3’) were used to detect the impact of expression of shorter RNAs (*csqR*-s). Primer positioning is shown in Supplementary Figure S3a. Promoters were mapped with the PlatProm algorithm^12,71^. No PCR products were detected in negative controls in the absence of reverse transcriptase. Data obtained from at least two biological samples and three statistical replicates were calculated by the ΔC_t_ method. The error bars indicate the standard deviations of corresponding mean values.

## Results

### Phylogenetic analysis of CsqR

**Figure 2:**
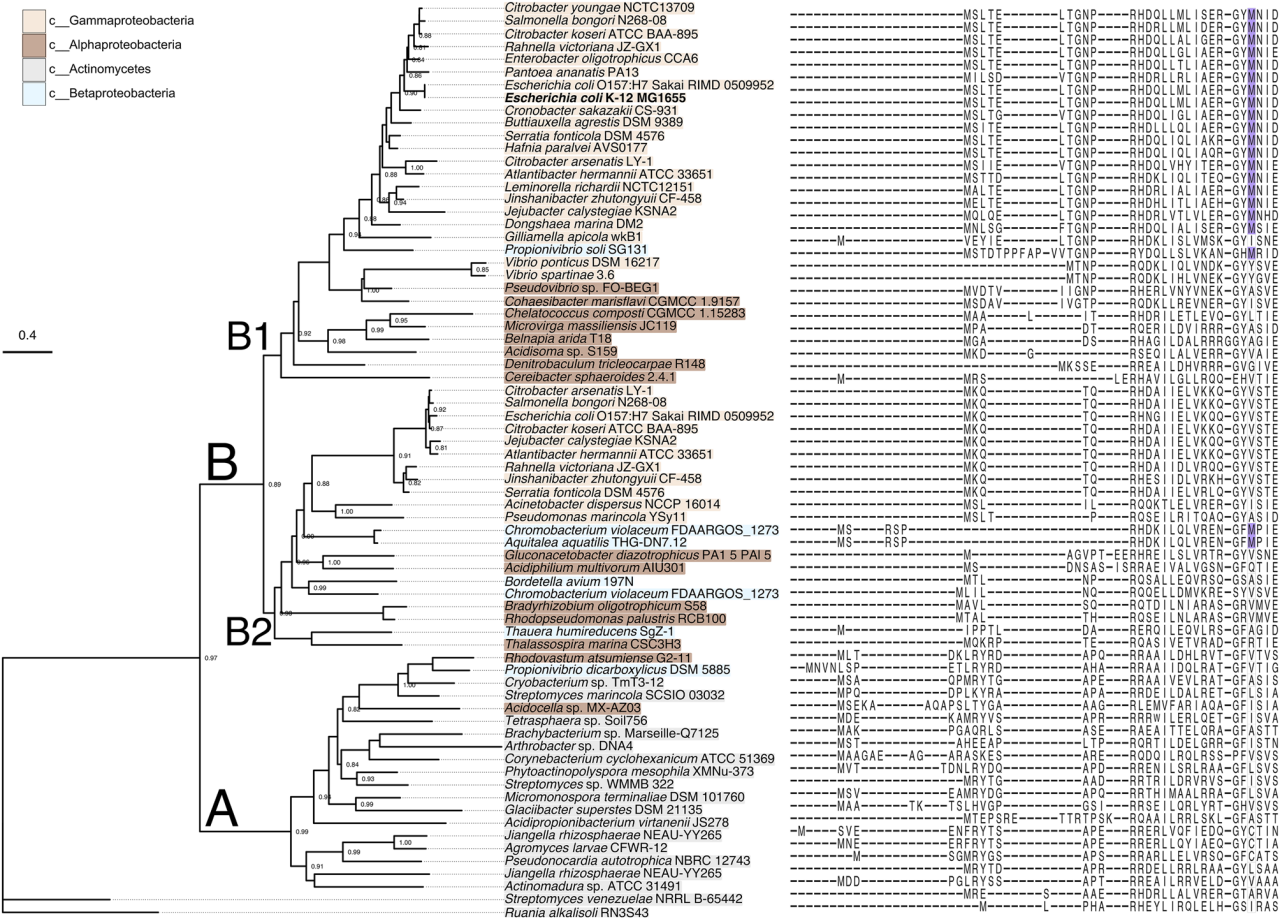
Phylogenetic tree of CsqR and its homologs from Gamma-, Beta-, Alphaproteobacteria and Actinomycetes. The left panel shows the tree inferred using the maximum likelihood algorithm and rooted manually by the branch leading to CsqR homologs from *Streptomyces venezuelae* str. NRRL B-65442 and *Ruania alkalisol*i str. RN3S43. The right panel features the first 62 positions of the multiple sequence alignment. Methionines aligned to Met25 of the reference *E. coli* str. K-12 MG1655 are marked violet.

The CsqR phylogenetic tree consists of two main clusters. One cluster contains CsqR homologs mainly from Actinobacteria (branch A) and another one corresponds to CsqR homologs from Proteobacteria (branch B) (Fig. 2). The Proteobacteria branch can be further divided into two groups. One is represented by close homologs of the reference CsqR from *E. coli* str. K-12 MG1655 (presumably orthologs) (Fig. 2, sub-branch B1). These proteins mainly come from Enterobacteriales and Vibrionales with some exceptions likely caused by horizontal gene transfer (Fig. 2, Supplementary Table S1). The second branch contains CsqR paralogs from Gammaproteobacteria and distant CsqR homologs from other classes of Proteobacteria (Fig. 2, Supplementary Table S1, sub-branch B2). The structure of the CsqR phylogenetic tree suggests that *csqR* might have been duplicated at some point (Fig. 2).

We observed highly conserved methionines at position 25 (Fig. 2). Met25 is present only in close homologs of CsqR from the branch B1 and is absent in *Vibrio* spp. (Fig. 2). In the Enterobacteriales branch, only CsqR of *Gilliamella apicola* str. wkB1 lacks Met25, which is substituted to isoleucine. Homologs of CsqR from the branch B2 lack Met25, with few exceptions (Betaproteobacteria *Chromobacterium violaceum* str. FDAARGOS_1273 and *Aquitalea aquatilis* str. THG-DN7.12) (Fig. 2). Most non-methionine residues aligned to Met25 are not encoded by alternative start codons GUG and UUG (Supplementary Table S1).

Conservation of Met25 across enterobacterial species points towards its functional significance. Therefore CsqR might be translated as two alternative forms, the common long one (CsqR-l) and the short one lacking 24 N-terminal residues (CsqR-s). Considering the distribution of Met25, CsqR-s might have originated in the common ancestor of Enterobacteriales.

Next we inspected the genomic context of the *csqR* gene in both branches. The co-localization pattern of the *csqR* gene mainly involves homologs of the *yih* locus genes in Proteobacteria (branch B1) and Actinobacteria (branch A) (Fig. 2, Supplementary Fig. S1). In particular, close *csqR* homologs from Gammaproteobacteria are co-localized with at least two *yih* locus genes, coding for kinase YihV and reductase YihU, with minor exclusions (Supplementary Fig. S1, branch B1). In Alphaproteobacteria and Actinobacteria, *csqR* homologs tend to cluster with *yihV* and *yihT* (aldolase). The genomic context of *csqR* might include genes from the sulfo-EMP2 gene cluster that is an alternative to the *yih* gene with the same function. It was observed in Actinobacteria, Alphaproteobacteria, and some Gammaproteobacteria species (Aeromonadaceae, Hafniaceae, Yersiniaceae, and Vibrionaceae). For instance, *slaB* (reductase) and *sqiK* (kinase) are likely substitutes for *yihU* and *yihV*, respectively, in *Micromonospora terminaliae* str. DSM 101760. The *sqgA* gene was found to substitute *yihQ* as sulfoquinovosidase in *Arthrobacter* sp. DNA4, and *sqvD* substituted *yihS* as isomerase in *Streptomyces* sp. WMMB 322. In *Hafnia paralvei* str. AVS0177 and *Serratia fonticola* str. DSM 4576, *sqvD* and *sqiA* replaced *yihS* and *yihT,* respectively.

According to the study by Sharma et al.^26^, *Hafnia paralvei* locus contains the *yihR* gene between *sqiA* and *sqvD*. Based on our data (Supplementary Fig. S1), the respective gene encodes aldolase but probably not YihR since its product (WP_004093390.1) and YihR from *E. coli* (AYG21325.1) share a relatively low sequence similarity (20.3% of identity and 28.3% of similarity). Same was observed for aldolase (WP_024484837.1) of *Serratia fonticola* (19.7% of identity and 31.9% of similarity)*.*

The co-localization pattern is not maintained for distant *csqR* genes homologs (likely paralogs) in some Gammaproteobacteria and in other classes of Proteobacteria (Supplementary Fig. S1, branch B2). Organisms with Met25 tend to harbor the *yih* locus genes in the respective genomic context.

### Expression of recombinant CsqR in *Escherichia coli*

**Figure 3:**
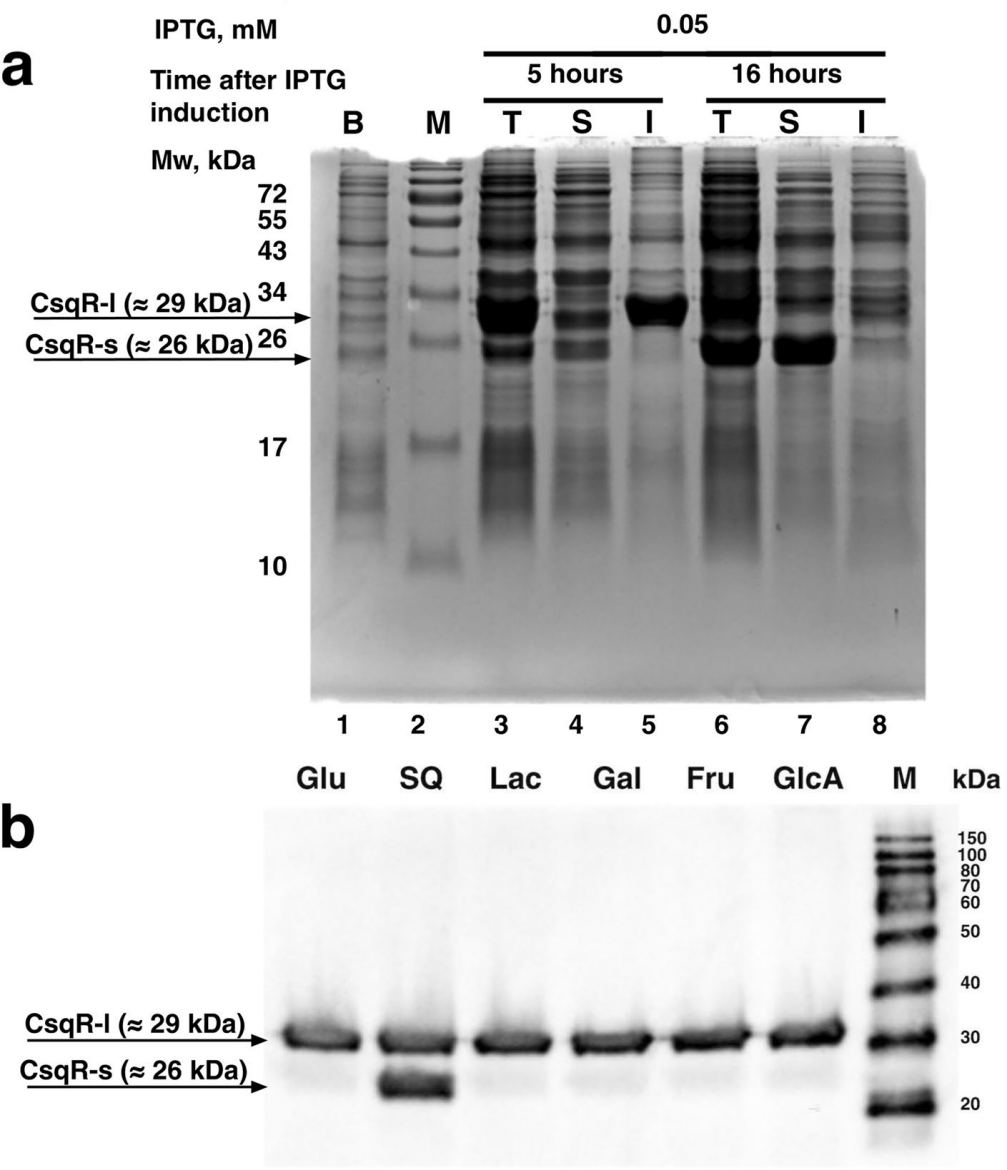
Detection of the short CsqR-s form. (**a**) Production of the recombinant CsqR protein in *E. coli* BL21 CodonPlus (DE3)-RIL after 5 and 16 h of growth on LB medium following IPTG induction at a final concentration of 0.05 mM. B—before induction, T—total cellular protein fraction, S—soluble fraction, I—insoluble fraction, M—protein molecular weight marker (Prestained Protein Marker #P7712, NEB). (**b**) Western blot analysis of CsqR-6xHis protein synthesis from the bacterial chromosome in *E. coli* str. K-12 MG1655. Above each line, the culture growth conditions are indicated: aerobic growth at 37˚C in the presence of 0.2% D-glucose (Glu), sulfoquinovose (SQ), lactose (Lac), D-galactose (Gal), D-fructose (Fru), or D-glucuronic acid (GlcA). M is the protein molecular weight marker (SuperSignal Molecular Weight Protein Ladder #84,785).

We then constructed the pGEM_YihW_TEV_his plasmid coding for CsqR with the 6xHis-tag on its C-terminal end to produce CsqR. Since transcription factors in high concentrations might be toxic for bacterial cells, we tested several strains and expression conditions (Supplementary Fig. S2), and the best result for production of the CsqR-l form was obtained in BL21 (DE3)-CodonPlus-RIL after 5 h of induction with 0.05 mM of IPTG (Fig. 3a, lane 5). The protein was in the insoluble fraction, but the minimal amount of trace proteins simplified further purification using one-step affinity chromatography.

After 16 h of induction CsqR-l almost disappeared, replaced by a shorter form of approximately 26 kDa (Fig. 3a, lanes 6 and 7). The size of this protein corresponds to CsqR-s translated from Met25.

### CsqR-s is actively expressed in the presence of sulfoquinovose

To validate the CsqR protein identity, we used the K-12 MG1655 strain bearing 6xHis-tag on the C-terminal end of CsqR in the chromosome and studied the respective protein production during growth with glucose, lactose, sulfoquinovose, galactose, fructose, and glucuronic acid after 4 h at 37 ˚C in aerobic conditions (Fig. 3b).

We observed a band of 29 kDa with similar intensity in all samples. It corresponded to the full-length CsqR-6xHis-tag protein (wild-type CsqR 28.507 kDa + 6x-His-tag 0.8 kDa), CsqR-l. We also detected a shorter form of about 26 kDa, CsqR-s, which was highly expressed in the presence of SQ and produced at low levels in other conditions (Fig. 3b).

To check observations on the mRNA level, qRT-PCR was done using two primer pairs (Supplementary Fig. S3a), differentially detecting expression of the full-sized *csqR*-mRNA (*csqR*-l) and the impact of the shorter RNAs from which CsqR-s can be translated. These shorter RNAs could be transcribed from the cluster of internal promoters predicted by PlatProm with rather low, but still sufficient scores of 4.2–4.7^12^. From Fig. S3b, it can be seen that *csqR* was activated during exponential growth with SQ, and that this activation was higher for *csqR*-s. Upon transition to the stationary phase, level of the *csqR*-l-mRNA on SQ was almost the same as on glucose, while *csqR*-s was still SQ-activated. This is in line with the dynamics of protein synthesis, with the CsqR-s form being prevalent during stationary growth (Fig. 3a).

No signal was detected when using either of the primers 1 or 2 (Supplementary Fig. S3a) with the second primer located upstream of yihWP1 (5’-TGATGTGGTAGATACCACAG-3’), suggesting that *csqR* (*yihW*) is transcribed independently from *yihV* and is subjected to its own regulation.

Since SQ had been shown to be an effector of CsqR^15^, and we here observed activation of CsqR-s during growth on this sugar, we next modeled both protein structures and performed docking with potential effectors.

### Molecular dynamic simulations of protein structures predicted for the long and short CsqR forms

To assess the structural properties of the CsqR forms, we obtained AlphaFold models for both products and compared them using flexible structural alignment (Supplementary Fig. S4).

In the predicted structure of each CsqR form, the N-terminal (NTD) and C-terminal domains (CTD) were suggested to be the DNA-binding and effector-sensing, respectively (Supplementary Figs. S4a, S5a, d). In general, for most positions in both AlphaFold CsqR models, pLDDT varied between confident (70 < pLDDT < 90) to high (pLDDT > 90) levels (Supplementary Fig. S4b), except for short C- and N- terminal segments in both predicted structures and the interdomain linker in CsqR-s (pLDDT < 50) (Supplementary Fig. S4b).

**Figure 4:**
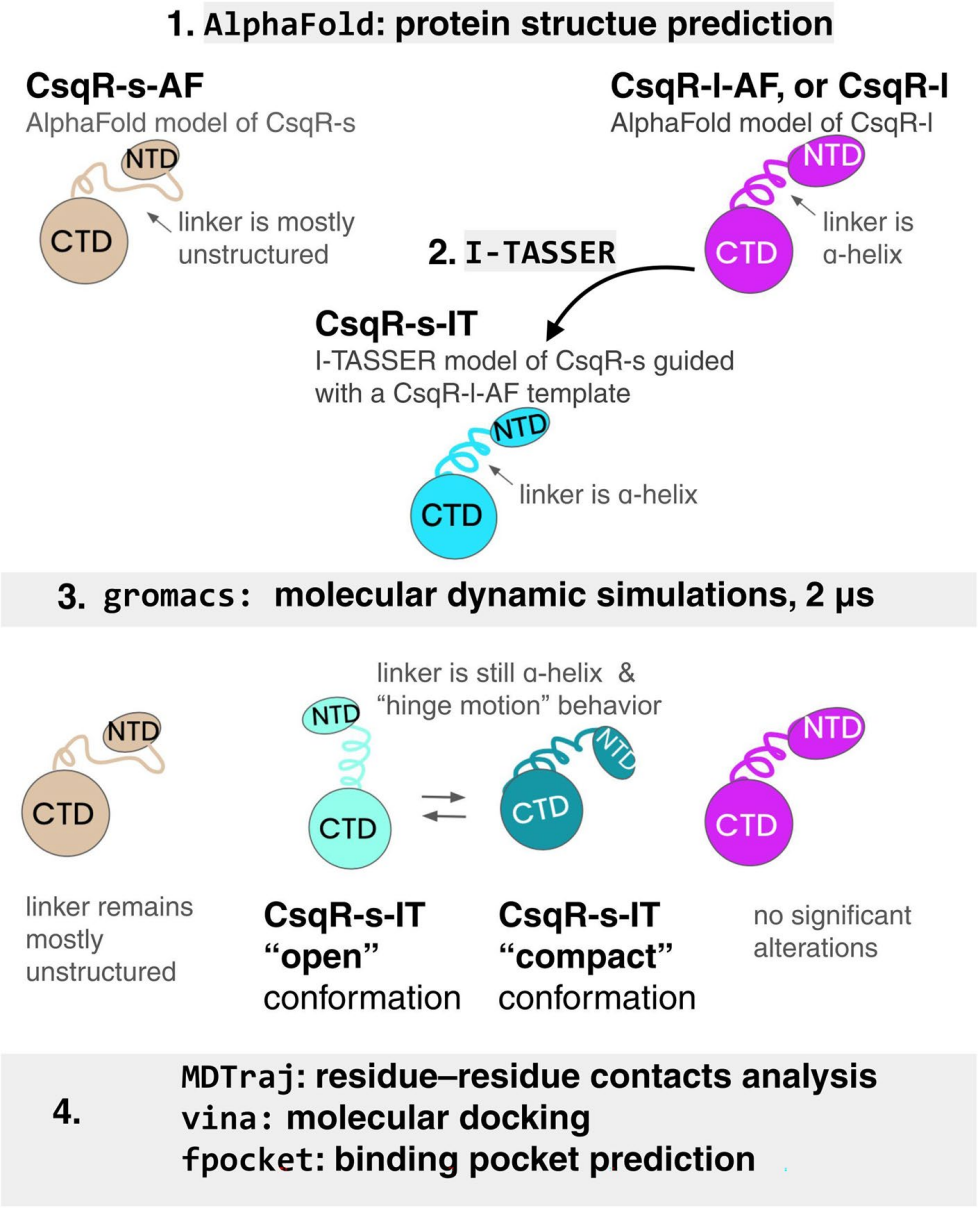
Schematic representation of the workflow used for structural modeling of CsqR. NTD—N-terminal domain, CTD—C-terminal domain. First, AlphaFold models were obtained for CsqR-s and CsqR-l (CsqR-s-AF and CsqR-l-AF models, respectively). The interdomain linker of CsqR-s-AF was predicted with a low confidence suggesting a loop region. To predict an alternative structure of CsqR-s with the more prominent structural arrangement of the interdomain linker, we obtained the I-TASSER model of CsqR-s using the CsqR-l-AF model as a template. The goal was to check if both types of CsqR-s models would fold to the similar structure of the linker after MDS.

To check if the interdomain linker of CsqR-s has a more pronounced structural arrangement, we used the following workflow (Fig. 4). First, we obtained an I-TASSER model of CsqR-s with the CsqR-l AlphaFold model used as a template (Supplementary Fig. S5g). Interdomain linker of this CsqR-s model was predicted as an ɑ-helix (Supplementary Fig. S5g). We then subjected the AlphaFold models of CsqR-l and CsqR-s (CsqR-l-AF and CsqR-s-AF, respectively) and the I-TASSER model of CsqR-s guided with a CsqR-l-AF template (CsqR-s-IT) to molecular dynamic simulations (MDS). The idea behind this setup has been to inspect what will happen with an interdomain linker after MDS, that is, whether it will become disordered in the CsqR-s-IT model and remain loop-like in CsqR-s-AF, or get an ɑ-helix arrangement in CsqR-s-AF and preserve an ɑ-helical structure in CsqR-s-IT (Fig. 4). After 2 µs of MDS, we extracted representative conformations from trajectories and analyzed their structural changes relative to the starting state (Fig. 4, Supplementary Fig. S5).

CsqR-l-AF did not alter its conformation significantly after MDS (Supplementary Fig. S5a–c). In the final model of CsqR-l, CTD had the conserved protein fold of the ISOCOT (isomerase, CoA transferase, and translation initiation factor) superfamily typical for the DeoR protein family^72^ (Supplementary Fig. S5a–c). It was confirmed by the structure similarity search using Dali^61^. Among the top hits were deoxyribose operon repressor DeoR (PDB ID: 7l6l-B; Z-score 26.6), ribose 5-phosphate isomerase RpiA (PDB ID: 4gmk-A; Z-score 16.0), and subunit ɑ of the translation initiation factor eIF-2B (PDB ID: 6jly-G; Z-score 14.6). The NTD of CsqR-l consisted of three ɑ-helices and a three-stranded β-sheet (Supplementary Fig. S5b), together arranged in a winged helix-turn-helix (wHTH) motif^73^. Such domain architecture is also common among DeoR-family regulators^74^.

**Figure 5:**
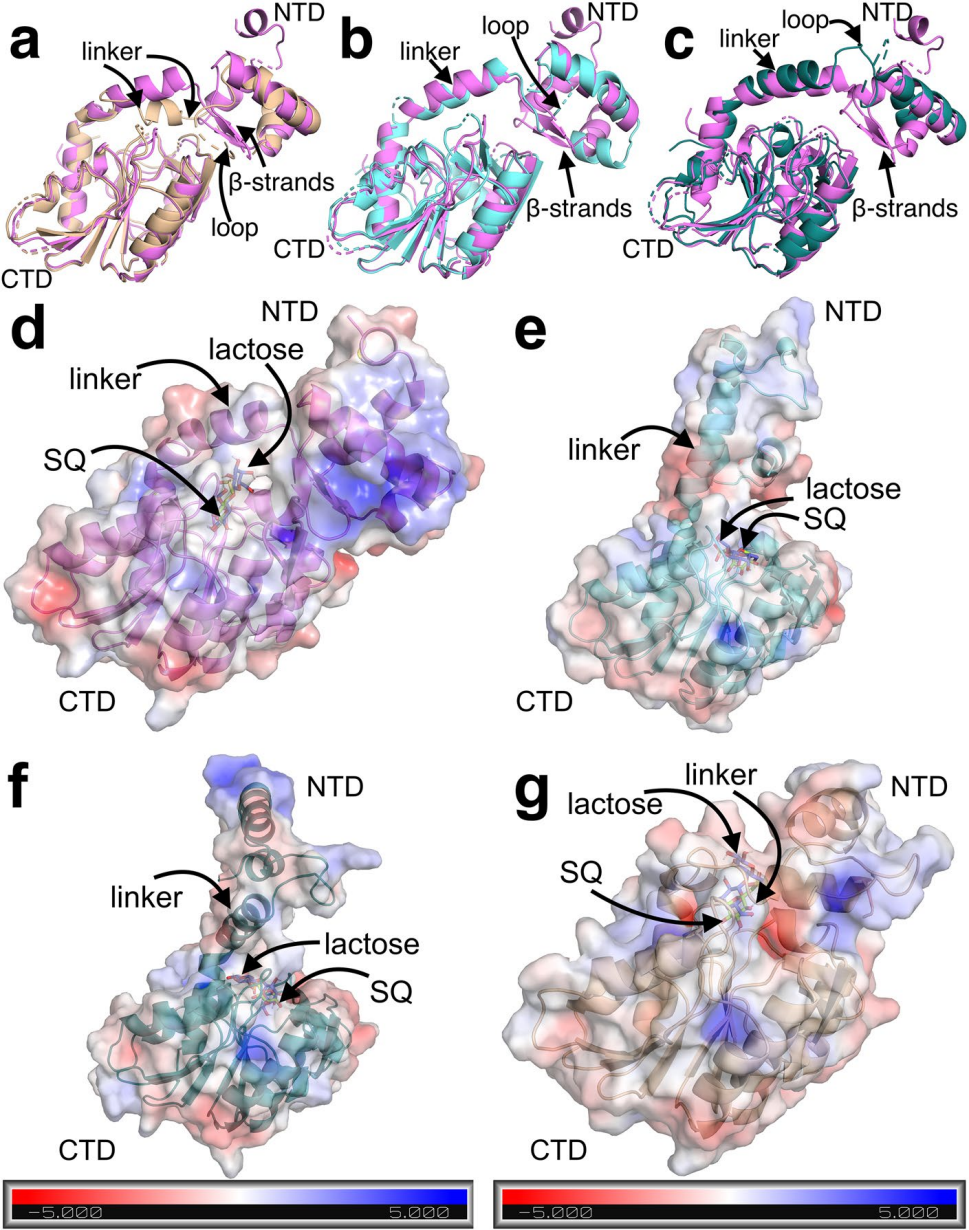
Structural modeling of CsqR. **(a–c).** Flexible structural alignment of CsqR-l (pink) and CsqR-s models. The main differences between the two structures are indicated with arrows. CsqR-l is pink, CsqR-s-AF is beige, CsqR-s-IT in the open conformation is light blue, CsqR-s-IT in the compact conformation is deep teal. Each CsqR-s model has the same orientation of the C-terminal domain as the CsqR-l model does. **(d–g)** Molecular docking of sulfoquinovose and lactose to CsqR-l (**d**), CsqR-s-IT in the open conformation (**e**), CsqR-s-IT in the compact conformation (**f**), and CsqR-s-AF (**g**). The best binding modes of the ligands are shown. Surfaces are colored according to the electrostatic potential values from negative red to positive blue. This result of molecular docking but from a different angle is provided in the Supplementary Figure S12. Results of molecular docking of CsqR-l and CsqR-s with SR, SQG, and glucose are shown in Supplementary Figure S13.

No notable differences were found in the CTDs of CsqR-s-AF and CsqR-s-IT compared to CsqR-l-AF (Fig. 5a–c). The NTDs of both final models of CsqR-s lacked an ɑ-helix and β-strands (Fig. 5a–c; Supplementary Fig. S6). The β-strands were initially predicted in NTD but turned into a coil-like structure after MDS (Fig. 5a–c, Supplementary Fig. S6). Another difference concerned the interdomain linker—the DNA-binding and ligand-binding domains of CsqR-l-AF were still connected by an ɑ-helix, while in CsqR-s-AF, the linker remained mostly unstructured, even after an additional 1 µs run of MDS with increased temperature of the protein (350 K) (Fig. 5a, Supplementary Figs. S5d–f, S6a, S7a). The NTD and CTD of the final CsqR-s-AF model got closer and shifted relative to each other (Supplementary Fig. S5d–f).

In CsqR-s-IT, the ɑ-helical interdomain linker did not get disrupted after 2 µs of MDS (Fig. 5b-c, Supplementary Figs. S5g–k, S6b). Moreover, we observed switching between two main conformations of CsqR-s-IT, “open” and “compact”, during MDS (Supplementary Figs. S5g–k, S8). In the open state, the interdomain ɑ-helix and the first ɑ-helix of the CTD formed a single ɑ-helix of about 27 residues long that separated NTD from CTD (Supplementary Figs. S5h, j; S6b). In the compact state, this long ɑ-helix bended, bringing NTD and CTD closer to each other (Supplementary Figs. S5i, k; S6b, S8). Such behavior was not observed for either CsqR-l-AF or CsqR-s-AF, as both had only one main conformation (Supplementary Fig. S5a–f).

To estimate which interatomic interactions might distinguish CsqR models and participate in their stabilization, we analyzed frequencies of residue-residue contacts in the trajectories comparing CsqR-s with CsqR-l (Supplementary Fig. S9).

CsqR-s-AF differed from CsqR-l mainly by the formation of new interactions between the NTD and CTD and the loss of several contacts within the interdomain linker (Supplementary Fig. S9a). This loss can be attributed to the disruption of the α-helix (Supplementary Figs. S9a, S10a–d). In particular, Asn69 of CsqR-s-AF started to form a hydrogen bond with Arg75 instead of Phe72 as it did in CsqR-l (Supplementary Fig. S10a–d). In CsqR-s-AF, Asn69 and Arg75 resided within the linker at the periphery of its loop region, hinting at their potential significance in preserving this loop through interaction with each other (Supplementary Fig. S10d). Due to the initial irregularity of the linker structure and following stabilization, the NTD began to form more contacts with the CTD during MDS (Supplementary Fig. S9a). Contacts between CTD and NTD differed by the involved NTD residues: in CsqR-l, it was Arg22 and His57, and in CsqR-s-AF, Arg63 and Ala64 (Supplementary Fig. S10e–h) interacting with a common region of CTD, namely, Ser219, His217, and Glu181 (Supplementary Fig. S10e–h). Additional hydrogen bonds between Glu29 and Ala241, specific for CsqR-s-AF, may contribute to the approximately 90-degree turn in the NTD position compared to that in CsqR-l (Supplementary Fig. S10h).

The comparison of the CsqR-l and CsqR-s-IT models yielded three main differences: (1) reorganization of contacts in the CTD due to different structural arrangement of its ɑ-helix (Supplementary Figs. S9c, d, S11e–h); (2) decrease in the number of contacts between the NTD and CTD (Supplementary Fig. S9c, d); and (3) rearrangement of hydrogen bonds at the boundary between the interdomain linker and the CTD (Supplementary Figs. S9c, d, S11a–d). The latter two differences were likely associated with the hinge-like rotation of the NTD in CsqR-s-IT. The key involved residues in CsqR-s-IT were Glu73, Glu76, Val77, Ser78 from the interdomain linker, and Glu81, Glu82, Lys83, and Arg129 from the CTD (Supplementary Figs. S9b–d, S11a–d). The bending of the interdomain α-helix towards the CTD could result from hydrogen bonds formed between the CTD residues Lys83 and Arg129, and the linker residues Glu76 (in CsqR-l and CsqR-s-IT compact), Arg75 (in CsqR-l), and Glu73 (in CsqR-s-IT compact) (Supplementary Figs. S9d, S11d). The motion of the NTD in CsqR-l might be constrained due to additional interactions between the NTD and CTD, particularly those involving Arg22 (Supplementary Figs. S9a, c, d, S10g). In the open conformation of CsqR-s-IT, rearranged hydrogen bonds of the linker residues caused the linker to disengage from Lys83 and Arg129, moving the NTD away from the CTD (Supplementary Fig. S11c). A 27-residue-long helix in the open CsqR-s-IT conformation was likely stabilized by interaction between the first ɑ-helix of the CTD (Glu81, Glu82) and the interdomain linker (Ser78, Val77) (Supplementary Figs. S9c, S11c). These linker residues and the CTD residues Lys83 and Arg129 are conserved among CsqR homologs (Supplementary Fig. S16).

### Molecular docking of CsqR-l and CsqR-s models with lactose, SQ and its derivatives

The absence of the ɑ-helix and β-strands in the NTD and the presence of the disordered loop between two structural domains might significantly affect the functioning of CsqR-s, including its ability to bind effectors. To address the possibility that both SQ and lactose might serve as CsqR effectors, we performed molecular docking to analyze the binding patterns of two CsqR forms (Fig. 5d–g, Supplementary Figs. S13, S14, S15, Table S2). We included SQG and SR in the analysis since they also might serve as putative effectors^15,18^ (Supplementary Figs. S13, S14, S15, Table S2). We used glucose as a control sugar as it had no influence on CsqR binding to the intergenic regions of the *yih* genes^15^.

Patterns of glucose docking were different in the CsqR-s and CsqR-l models. In CsqR-l, the glucose binding site was primarily predicted at the same location as for other ligands with binding affinity comparable to that of SQ and its derivatives (Fig. 5d, Supplementary Figs. S13a–b, S14). Among candidate effectors, only lactose demonstrated higher binding affinity towards CsqR-l than glucose (Supplementary Fig. S14).

In CsqR-s-IT models, optimal docking positions of glucose were mostly different from those of candidate effectors while in CsqR-s-AF, glucose was predominantly docked to the same pocket as other ligands (Supplementary Fig. S13c–h). On average, in CsqR-s, glucose had significantly less effective energy of binding compared to lactose, SQ, SR, and SQG, regardless of the interdomain linker structure (Supplementary Fig. S14). We may therefore suggest that CsqR-s has higher specificity towards candidate effectors than CsqR-l.

On average, all CsqR models demonstrated the highest binding affinity towards lactose. The affinities for SQ and SR were similar to each other, while the affinity for SQG was slightly lower (Supplementary Fig. S14). Compact and open conformations of CsqR-s-IT did not differ significantly in the binding energy and, in general, had lower predicted affinity towards ligands than CsqR-s-AF and CsqR-l-AF (Supplementary Fig. S14).

**Figure 6:**
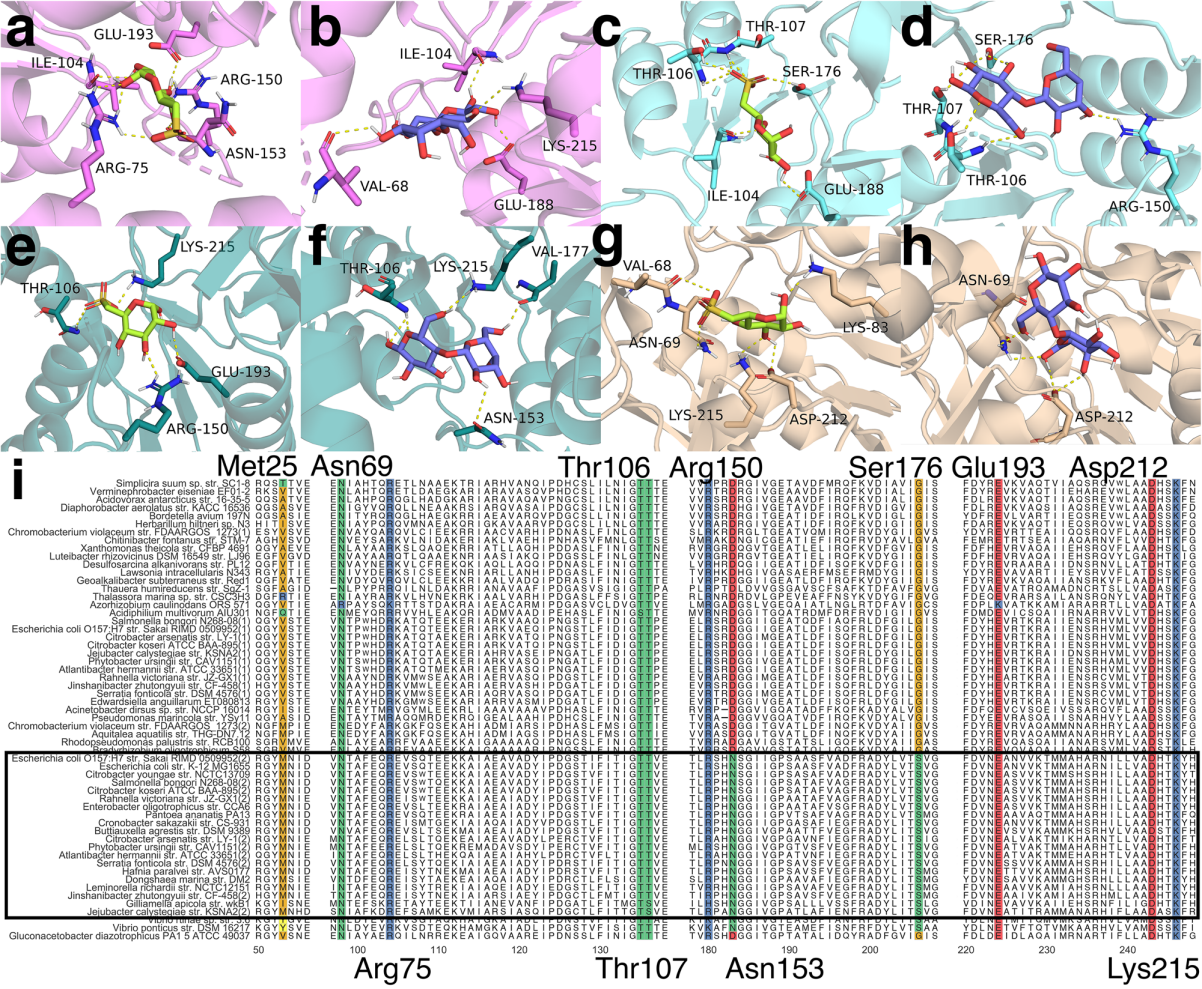
Ligand binding sites predicted for CsqR. (**a–h**) Ligand binding sites of CsqR models were predicted by AutoDock Vina. Within each protein–ligand complex, one of optimal ligand modes is shown: (**a**) CsqR-l and SQ; (**b**) CsqR-l and lactose; (**c**) CsqR-s-IT (open) and SQ; (**d**) CsqR-s-IT (open) and lactose; (**e**) CsqR-s-IT (compact) and SQ; (**f**) CsqR-s-IT (compact) and lactose; (**g**) CsqR-s-AF and SQ; (**h**) CsqR-s-AF and lactose. Polar contacts are marked by a dashed yellow line. Ligand binding sites of CsqR with SR and SQG are provided in Supplementary Figure S15. (**i**) Multiple sequence alignment of CsqR homologs. A part of the alignment (positions 50–56, 97–138, 178–208, 220–248) is shown. CsqR homologs with Met25 are in the black frame. Positions mentioned in the text are highlighted: Met25, Asn69, Arg75, Thr106, Thr107, Arg150, Asn153, Ser176, Glu193, Asp212, and Lys215. Numbering of amino acid residues in the CsqR-s is according to CsqR-l.

In each CsqR model, optimal binding modes for lactose, SQ, and its derivatives were all located within a pocket, with the positively charged part of the pocket affecting the orientation of the sulfonic acid group of sulfonated ligands (Fig. 5d–g, Supplementary Fig. S13). In CsqR-l and CsqR-s-IT (both conformations), the pocket was formed by the CTD and the interdomain linker (Fig. 5d–f, Supplementary Fig. S13a, b, e–h). The same set of amino-acid residues was involved in polar contacts with ligands in CsqR-l and CsqR-s-IT (Fig. 6a–f, Supplementary Fig. S15a–f). These residues included Arg150, Glu193, Lys215, Asn153, and Ser176 (Fig. 6a–f, Supplementary Fig. S15a–f). The residues Arg150, Glu193, Lys215 are highly conserved among homologs of CsqR (Fig. 6i, Supplementary Fig. S16). Moreover, Asn153 and Ser176 are present only in those CsqR homologs that also possess Met25 (Fig. 6i). Other conserved residues were found in the ligand binding site of CsqR-l, such as Arg75, or featured in the ligand positioning in CsqR-s-IT like Thr106 and Th107 (Fig. 6a–f, i, Supplementary Figs. S15a–f, S16).

In CsqR-s-AF, the ligand binding site was shifted to the interdomain interface, and the flexible linker participated in the ligand positioning (Fig. 5g, Supplementary Fig. S13c, d). Lys215 was the common residue for all CsqR models, including CsqR-s-AF, predicted to interact with lactose, SQ, SR, and SQG (Fig. 6g–i, Supplementary Fig. S15g, h). Residues specific for the ligand binding sites of CsqR-s-AF are Asn69 from the flexible linker and Asp212 from the CTD, both highly conserved in CsqR (Fig. 6i, Supplementary Fig. S16).

To compare positions of the residues predicted in the ligand binding sites, we rigidly aligned each CsqR-s model to CsqR-l and calculated all-atom and backbone RMSD for the respective residues (Supplementary Fig. S17). Residues with RMSD values less than 3 Å were considered spatially similar^75^. The residues in the CTD were structurally close (Supplementary Fig. S17). Only Asn153 from the CTD had slightly higher RMSD than 3 Å, as expected from the residue located in a flexible loop (Supplementary Fig. S17). Location of the linker residues Asn69 and Arg75 differed significantly probably due to rearrangement of hydrogen bonds in the linker of the CsqR-s models (Supplementary Figs. S17, S10a–d, S11a–d).

CsqR-s-IT had a different orientation of the interdomain linker compared to CsqR-l (Supplementary Fig. S18e, f, i, j) which might be associated with a decrease in the number of contacts between the NTD and CTD (Supplementary Fig. S9c–d). We may suggest that due to this alteration in the linker position, the boundaries of the binding pocket in CsqR-s-IT changed, leading to the involvement of Thr106 (both open and compact CsqR-s-IT) and Thr107 (open CsqR-s-IT) in the ligand binding (Supplementary Fig. S18e–l). At the same time, Arg75 moved out of the binding pocket in CsqR-s-IT, losing interaction with the CTD residue Lys83 (Supplementary Figs. S18h, l, S11c, d). Instead, Arg75 formed new polar contacts with residues from the linker and NTD in the compact conformation, or solely within the linker in the open conformation (Supplementary Fig. S11c, d).

The unstructured interdomain linker in CsqR-s-AF likely required more contacts between the NTD and CTD for protein stability (Supplementary Figs. S9a, S10e–h), resulting in a positional shift of the NTD relative to the CTD (Supplementary Fig. S18a–d). Consequently, the binding pocket of CsqR-s-AF expanded (Supplementary Fig. S18a–d). The optimal docking mode moved towards Asp212, which became involved in the ligand binding (Supplementary Fig. S18a–d). Asn69 rearranged its hydrogen bonds within the linker, in particular it started to interact with Arg75, probably maintaining the loop part of the linker (Supplementary Fig. S10a–d). Orientation of Asn69 and Arg75 changed relative to the respective one in CsqR-l so that Arg75 did not longer participate in the ligand binding in CsqR-s-AF while Asn69, on the contrary, entered the binding pocket of CsqR-s-AF and began to interact with ligands (Supplementary Fig. S18a–d).

## Discussion

Despite recent advances in understanding the regulatory role of CsqR, little is known about its evolution and structural properties, including its interactions with effector molecules.

Recently, Duarte-Velázquez et al*.*^23^ suggested either gene duplication or horizontal gene transfer as the main factors driving evolution of several transcription regulators in *E. coli*, including CsqR. Our observation on the phylogeny of CsqR extends and clarifies this assumption (Fig. 2). In some Actinobacteria, Alpha- and Gammaprotebacteria species, gene neighborhood of *csqR* contains a mosaic of homologs of the *yih* cassette genes and genes from the sulfo-EMP2 locus in a mutually exclusive way which is probably a result of non-orthologous gene displacement^76^.

Many *csqR* homologs possess a conserved AUG codon at position 25 downstream of the main start, indicating its functional importance (Fig. 2). Thus, CsqR could have an alternative protein form truncated at the N-terminus, potentially originating in a common ancestor of Enterobacteriales (Fig. 2). Such *csqR* genes tend to be co-localized with homologs of genes from the *yih* cassette, pointing to a specific, functional link between the CsqR-s form and the *yih* cassette (Fig. 2, Supplementary Fig. S1). Since the *yih* locus is mainly present in Enterobacteriales (Supplementary Fig. S1), we assume that the alternative start could have appeared together with the *yih* cassette.

While producing the recombinant CsqR, we indeed observed synthesis of the protein with Mw of around 26 kDa (Fig. 3a) that could be the shortened CsqR form. This short protein tends to accumulate with time, becoming prevalent after 16 h of growth (Fig. 3a). Western blot analysis confirmed that the detected short variant may indeed be the shorter version of CsqR-l, translated in-frame, and in the used conditions its synthesis was activated in response to SQ (Fig. 3b). During growth with other carbon sources, CsqR-s was detected in minor quantities (Fig. 3b).

Based on the presence of alternative conserved methionines and additional intragenic promoters for synthesis of shortened mRNAs (Supplementary Fig. S3) we suggest that each variant of CsqR could arise via translation initiation at different start codons. An alternative hypothesis might be that CsqR-s is a result of the CsqR-l proteolysis. However, taking into account the expression dynamics of the respective mRNAs (Supplementary Fig. S3) being in line with the protein levels (Fig. 3), this is much less prominent.

Given that both *csqR*-s mRNA and the CsqR-s protein levels are enhanced in response to SQ (Supplementary Fig. S3b), especially at the stationary phase (Supplementary Fig. S3b), this might suggest that CsqR-s is needed when the rich carbon source becomes depleted. This is in line with production of CsqR-s at the stationary phase (Fig. 3). At the exponential phase, in turn, the main form CsqR-l is needed for normal functioning of the related metabolic pathways. Upon transition to the stationary growth, CsqR-s begins to be synthesized and could act as an inhibitor of CsqR-l.

The role of SQ as an effector for CsqR was recently investigated by Shimada et al.^15^. Using the gel shift assay, they showed that SQ might decrease the efficiency of CsqR binding to its targets, including the *csqR* promoter region. They proposed a model where SQ might contribute to de-repression of *csqR* by inducing dissociation of CsqR from the *csqR* regulatory region. Our observation on the probable inducer role of SQ in the production of CsqR-s is consistent with this hypothetical scheme. Significant production of CsqR-s, but not of CsqR-l in the presence of inducer SQ, together with its dominant synthesis during the stationary phase of growth might reflect the possible regulatory role of CsqR-s under starvation.

To investigate binding properties of CsqR towards SQ and lactose, we modeled the structures of both CsqR forms. The AlphaFold and I-TASSER models of CsqR-s do not contain the first ɑ-helix and β-strands of the N-terminal DeoR-type wHTH motif, in contrast to CsqR-l (Fig. 5a–c, Supplementary Fig. S6). The first helix might be involved in non-specific binding to DNA^16^. The two other helices, common for both CsqR models, determine DNA-binding specificity, in particular the third helix that participates in specific interactions with the DNA major groove^77^. Therefore, it is possible that CsqR-s might recognize the same binding sites, albeit with lower affinity towards DNA.

According to docking results, candidate ligand binding sites of CsqR included residues from the interdomain linker and the CTD (Supplementary Fig. S17). The NTD did not participate in ligand binding directly. At the same time, there might be indirect effects of the NTD on ligand binding. Truncated NTD might establish more contacts with the CTD in case of an unstructured linker (the CsqR-s-AF model) (Supplementary Figs. S9a, S10e–h), or stop contacting the CTD if a linker exhibited a hinge motion behavior (CsqR-s-IT models) (Supplementary Figs. S9c, d, S18e–l). As a result, the volume of the binding pocket together with optimal docking positions might change (Supplementary Fig. S18). The NTD truncation may lead to a greater ability to differentiate between candidate effectors and other compounds compared to CsqR-l (Supplementary Figs. S14, S13e–h).

Molecular dynamic simulations provided evidence for two possible mechanisms for stabilizing the structure of CsqR-s: the interdomain linker can either be disordered (CsqR-s-AF model) or remain in the ɑ-helical arrangement (CsqR-s-IT model). If the domains are connected via the ɑ-helix, NTD might obtain ability to rotate in a hinge motion relative to CTD. Two conformational states may arise with the same ligand binding sites located in CTD (Supplementary Fig. S5g–k, Fig. 5e–f, Supplementary Figs. S8, S13e–h). Such a flexion behavior of the linker in CsqR-s might be essential for modulating its interaction with DNA when the regulator is in an oligomeric state. For example, YvoA, a GntR/HutC transcription factor from *Bacillus subtilis*, exhibits a pivot-like motion similar to that of the CsqR-s-IT model^78^.YvoA functions as a homodimer. Upon binding effector N-acetylglucosamine-6-phosphate, the interdomain region switches from a loop to a helix. As a result, NTDs of homodimer rotate and reorient apart in the ‘jumping jack’-like motion. After that, YvoA releases the DNA region leading to de-repression of genes under its regulation. Interestingly, the DNA-bound and effector-bound modes of YvoA resemble the open and compact conformations of the CsqR-s-IT model, respectively.

Analysis of the predicted ligand binding sites shows that the Asn69 residue located in the interdomain loop of CsqR-s-AF forms polar contacts with lactose and SQ, SR, and SQG (Fig. 6g–h, Supplementary Fig. S15g–h). In LacI, Asp149 positioned at the beginning of the flexible linker interacts with the inducer and participates in propagating the binding signal from the pocket to the N-terminal part of the protein^79^. Similarly, Asn69 of CsqR-s might participate in a structural transition induced by the effector. Interestingly, Asn69 usually occurs in proteins whose genes might encode the short form arising at the second translation start, hence pointing towards its specific importance for functioning of CsqR-s (Fig. 6i).

Asp212 and Lys215 also formed polar contacts with candidate ligands (Fig. 6g–h, Supplementary Fig. S15gh). They are conserved in other DeoR-type regulators^19^ and present in UlaR of *E. coli* (Asp206 and Lys209)^19^ and in LacR of *Lactococcus lactis* (Asp210 and Lys213)^80^. Site-directed mutagenesis and gel shift assay demonstrated that Asp210 and Lys213 of LacR are necessary for binding its inducer tagatose-6-phosphate^80^. Similarly, it was shown that Asp206 and Lys209 of UlaR directly participate in binding its effector molecule L-ascorbate-6-phosphate^19^.

In any scenario, whether in the compact and open states, or with the unstructured interdomain linker, CsqR-s might discriminate effectors from other compounds better than CsqR-l (Supplementary Figs. S14, S13e–h). This could be due to differences in the binding affinity (CsqR-s-AF) (Supplementary Fig. S14), binding site location, or both (CsqR-s-IT) (Supplementary Figs. S14, S13e–h). In CsqR-l, only lactose was predicted to exhibit higher affinity to the protein compared to glucose that was taken as the internal control (Supplementary Fig. S14), while CsqR-s could also use all tested sulfonated sugars (SQ, SR and SQG) as potential ligands. Thus, production of CsqR-s might be more crucial for regulating the *yih* genes during growth on SQ than on lactose which is in line with the western blot analysis (Fig. 3b).

Bacterial transcriptional regulators with several forms are known, but rare. The first reported example is VirF of *Shigella* spp. belonging to the AraC family^81^. We have observed that LeuO of *Escherichia coli*, a transcriptional regulator from the LysR family, is synthesized in several protein forms^82^. CsqR (YihW) is an interesting addition to this small set, ripe for further experimental analysis.

### Supplementary Information

Supplementary Information 1.

Supplementary Information 2.

## Data Availability

Additional files and code can be found in the GitHub at https://github.com/rybinaanya/YihW.
