# Supplementary material for: Phylogeny and structural modeling of the transcription factor CsqR (YihW) from Escherichia coli
Source: Sci Rep. 2024 Apr 3;14:7852. doi: 10.1038/s41598-024-58492-y (PMC10991401; doi:10.1038/s41598-024-58492-y)
Supplement: Supplementary file 1 — Supplementary Information 1. [file 41598_2024_58492_MOESM1_ESM.pdf]

Phylogeny and structural modeling of the transcription factor CsqR (YihW) from *Escherichia coli*

Anna A. Rybina[1*], Roman A. Glushak[2], Tatiana A. Bessonova[3], Artemiy I. Dakhnovets[1], Alexander Yu. Rudenko[4], Ratislav M. Ozhiganov[4], Anna D. Kaznadzey[5], Maria N. Tutukina[1,3,5], Mikhail S. Gelfand[1, 5]

[1]Skolkovo Institute of Science and Technology, Moscow, 121205, Russia

[2]Faculty of Biology, Lomonosov Moscow State University, Moscow, 119234, Russia

[3]Institute of Cell Biophysics RAS (Federal Research Center "Pushchino Scientific Center for Biological Research RAS"), Pushchino, 142290, Russia

[4]Belozersky Institute of Physico-Chemical Biology, Lomonosov Moscow State University, Moscow, 119991, Russia

[5]Institute for Information Transmission Problems RAS, Moscow, 127051, Russia

*rybinaaann@gmail.com

Supplementary Information

Table S1. Homologs of CsqR (YihW) used to construct the phylogenetic tree.

Full organism name	Assembly accession version	Class	RefSeq protein id	Identity %	Amino-acid residue aligned to Met25 of *E. coli*	Respective codon
Dongshaea marina DM2	GCF_003072645.1	Gammaproteobacteria	WP_108650252.1	68.482	M	AUG
Jejubacter calystegiae KSNA2	GCF_005671395.1	Gammaproteobacteria	WP_138096356.1	60.769	M	AUG
Denitrobaculum tricleocarpae R148	GCF_007004665.1	Alphaproteobacteria	WP_142899747.1	47.6	G	GGG
Acidipropionibacterium virtanenii JS278	GCF_003325455.1	Actinomycetes	WP_114043810.1	28.063	A	GCC
Rahnella victoriana JZ-GX1	GCF_021276285.1	Gammaproteobacteria	WP_095924333.1	85.098	M	AUG
Belnapia arida T18	GCF_016773205.1	Alphaproteobacteria	WP_202833638.1	38.956	A	GCC
Salmonella bongori N268-08	GCF_000439255.1	Gammaproteobacteria	WP_000815083.1	45.528	V	GUC
Thauera humireducens SgZ-1	GCF_001051995.2	Betaproteobacteria	WP_048708909.1	40.693	A	GCC
Acidiphilium multivorum AIU301	GCF_000202835.1	Alphaproteobacteria	WP_007422904.1	41.41	Q	CAG
Tetrasphaera sp. Soil756	GCF_001428065.1_Soil756	Actinomycetes	WP_162254369.1	29.482	I	AUC
Buttiauxella agrestis DSM 9389	GCF_013234275.1	Gammaproteobacteria	WP_172896013.1	77.132	M	AUG
Propionivibrio dicarboxylicus DSM 5885	GCF_900099695.1_IMG-taxon_2599185173_annotated_assembly	Betaproteobacteria	WP_091937510.1	26.25	V	GUC

Full organism name	Assembly accession version	Class	RefSeq protein id	Identity %	Amino-acid residue aligned to Met25 of *E. coli*	Respective codon
Microvirga massiliensis JC119	GCF_001006805.1_Microvirga_massiliensis_JC119	Alphaproteobacteria	WP_048709493.1	42.292	A	GCG
Streptomyces marincola SCSIO 03032	GCF_002128305.1	Actinomycetes	WP_086161815.1	25.6	L	CUG
Citrobacter koseri ATCC BAA-895	GCF_000018045.1	Gammaproteobacteria	WP_012133927.1	89.02	M	AUG
Citrobacter koseri ATCC BAA-895	GCF_000018045.1	Gammaproteobacteria	WP_012135527.1	44.309	V	GUC
Jiangella rhizosphaerae NEAU-YY265	GCF_003579925.1	Actinomycetes	WP_158602919.1	32.093	L	CUG
Citrobacter arsenatis LY-1	GCF_004353845.1	Gammaproteobacteria	WP_008786989.1	71.154	M	AUG
Citrobacter arsenatis LY-1	GCF_004353845.1	Gammaproteobacteria	WP_003023499.1	43.902	V	GUC
Cryobacterium sp. TmT3-12	GCF_004403255.1	Actinomycetes	WP_134361985.1	30.502	A	GCC
Escherichia coli O157:H7 Sakai RIMD 0509952	GCF_000008865.2	Gammaproteobacteria	NP_312293.1	44.715	V	GUC
Jinshanibacter zhutongyuii CF-458	GCF_004295645.1	Gammaproteobacteria	WP_130593208.1	43.32	V	GUG
Citrobacter youngae NCTC13709	GCF_900638065.1_55685_A01	Gammaproteobacteria	WP_003847907.1	87.786	M	AUG
Phytoactinopolyspora mesophila XMNu-373	GCF_010122465.1	Actinomycetes	WP_246220629.1	27.344	L	CUC
Jejubacter calystegiae KSNA2	GCF_005671395.1	Gammaproteobacteria	WP_138094583.1	43.777	V	GUC
Gilliamella apicola wkB1	GCF_000599985.1	Gammaproteobacteria	WP_202961627.1	59.494	I	AUU

Full organism name	Assembly accession version	Class	RefSeq protein id	Identity %	Amino-acid residue aligned to Met25 of *E. coli*	Respective codon
Micromonospora terminaliae DSM 101760	GCF_009671205.1	Actinomycetes	WP_154225529.1	28.75	L	CUC
Acidocella sp. MX-AZ03	GCF_027626035.1	Alphaproteobacteria	WP_008493785.1	27.451	I	AUU
Streptomyces sp. WMMB 322	GCF_900090145.1_IMG-taxon_2522125135_annotated_assembly	Actinomycetes	WP_074467019.1	28.049	I	AUC
Rhodopseudomonas palustris RCB100	GCF_016584445.1	Alphaproteobacteria	WP_011159945.1	41.633	V	GUG
Glaciibacter superstes DSM 21135	GCF_000421145.1	Actinomycetes	WP_169515662.1	27.49	V	GUC
Brachybacterium sp. Marseille-Q7125	GCF_023062615.1	Actinomycetes	WP_246958063.1	25.896	A	GCG
Propionivibrio soli SG131	GCF_025770765.1	Betaproteobacteria	WP_263770994.1	55.378	M	AUG
Pantoea ananatis PA13	GCF_000233595.1	Gammaproteobacteria	WP_141118340.1	81.569	M	AUG
Pseudomonas marincola YSy11	GCF_900682675.2_P_marincola_YSy11_v2	Gammaproteobacteria	WP_150548723.1	40.0	A	GCC
Pseudovibrio sp. FO-BEG1	GCF_000236645.1	Alphaproteobacteria	WP_008547099.1	54.98	A	GCC
Pseudonocardia autotrophica NBRC 12743	GCF_003945385.1	Actinomycetes	WP_085916552.1	28.054	C	UGU
Bradyrhizobium oligotrophicum S58	GCF_000344805.1	Alphaproteobacteria	WP_015668717.1	42.857	V	GUC
Streptomyces venezuelae NRRL B-65442	GCF_001886595.1	Actinomycetes	WP_015035635.1	51.02	A	GCC
Escherichia coli K-12 MG1655	GCF_000005845.2	Gammaproteobacteria	NP_418320.2	100.0	M	AUG

Full organism name	Assembly accession version	Class	RefSeq protein id	Identity %	Amino-acid residue aligned to Met25 of *E. coli*	Respective codon
Escherichia coli O157:H7 Sakai RIMD 0509952	GCF_000008865.2	Gammaproteobacteria	NP_312834.2	100.0	M	AUG
Bordetella avium 197N	GCF_000070465.1	Betaproteobacteria	WP_012417676.1	40.4	A	GCC
Gluconacetobacter diazotrophicus PA1 5 PAI 5	GCF_000021325.1	Alphaproteobacteria	WP_012225256.1	44.672	V	GUC
Salmonella bongori N268-08	GCF_000439255.1	Gammaproteobacteria	WP_000059686.1	89.02	M	AUG
Cereibacter sphaeroides 2.4.1	GCF_000273405.1 _Rhod_Spha_2_4_ 1_V1	Alphaproteobacteria	WP_023003416.1	35.371	V	GUC
Enterobacter oligotrophicus CCA6	GCF_009176645.1	Gammaproteobacteria	WP_152082866.1	87.8423	M	AUG
Vibrio ponticus DSM 16217	GCF_009938225.1	Gammaproteobacteria	WP_083627253.1	43.548	Y	UAC
Corynebacterium cyclohexanicum ATCC 51369	GCF_020886775.1	Actinomycetes	WP_229231008.1	29.555	V	GUC
Chelatococcus composti CGMCC 1.15283	GCF_014641535.1	Alphaproteobacteria	WP_183335043.1	40.4	L	CUG
Serratia fonticola DSM 4576	GCF_001006005.1	Gammaproteobacteria	WP_024484841.1	80.469	M	AUG
Serratia fonticola DSM 4576	GCF_001006005.1	Gammaproteobacteria	WP_021181740.1	41.296	V	GUC
Thalassospira marina CSC3H3	GCF_002844375.1	Alphaproteobacteria	WP_101271738.1	40.239	R	CGC
Cronobacter sakazakii CS-931	GCF_003516125.1	Gammaproteobacteria	WP_007887565.1	80.228	M	AUG
Acinetobacter dispersus NCCP 16014	GCF_009884975.1	Gammaproteobacteria	WP_005184250.1	42.742	I	AUC

Full organism name	Assembly accession version	Class	RefSeq protein id	Identity %	Amino-acid residue aligned to Met25 of *E. coli*	Respective codon
Atlantibacter hermannii ATCC 33651	GCF_008064855.1	Gammaproteobacteria	WP_002436481.1	43.902	V	GUC
Ruania alkalisoli RN3S43	GCF_014960965.1	Actinomycetes	WP_193496802.1	45.098	I	AUC
Chromobacterium violaceum FDAARGOS_1273	GCF_016890085.1	Betaproteobacteria	WP_043613317.1	41.339	M	AUG
Arthrobacter sp. DNA4	GCF_024362385.1	Actinomycetes	WP_255174621.1	26.908	I	AUU
Cohaesibacter marisflavi CGMCC 1.9157	GCF_900115225.1_IMG-taxon_2617270874_annotated_assembly	Alphaproteobacteria	WP_090075412.1	51.562	I	AUC
Actinomadura sp. ATCC 31491	GCF_022664455.2	Actinomycetes	WP_242375664.1	27.876	V	GUC
Acidisoma sp. S159	GCF_009766125.1	Alphaproteobacteria	WP_159014731.1	40.891	V	GUC
Rhodovastum atsumiense G2-11	GCF_937425535.1_Rhodovastum_atsumiense_G2-11	Alphaproteobacteria	WP_162530533.1	28.87	V	GUG
Jiangella rhizosphaerae NEAU-YY265	GCF_003579925.1	Actinomycetes	WP_119663033.1	27.309	C	UGC
Chromobacterium violaceum FDAARGOS_1273	GCF_016890085.1	Betaproteobacteria	WP_043613346.1	41.322	V	GUC
Rahnella victoriana JZ-GX1	GCF_021276285.1	Gammaproteobacteria	WP_095923856.1	44.872	V	GUC
Aquitalea aquatilis THG-DN7.12	GCF_005155025.1	Betaproteobacteria	WP_059286917.1	43.254	M	AUG
Leminorella richardii NCTC12151	GCF_900478135.1_28193_H01	Gammaproteobacteria	WP_111739404.1	75.197	M	AUG
Jinshanibacter zhutongyuii CF-458	GCF_004295645.1	Gammaproteobacteria	WP_130592469.1	74.016	M	AUG
Atlantibacter hermannii ATCC 33651	GCF_008064855.1	Gammaproteobacteria	WP_002436639.1	68.217	M	AUG
Vibrio spartinae 3.6	GCF_014083925.1	Gammaproteobacteria	WP_182287873.1	44.355	Y	UAC

Full organism name	Assembly accession version	Class	RefSeq protein id	Identity %	Amino-acid residue aligned to Met25 of *E. coli*	Respective codon
Agromyces larvae CFWR-12	GCF_022811705.1	Actinomycetes	WP_243558372.1	30.677	C	UGC
Hafnia paralvei AVS0177	GCF_020150375.1	Gammaproteobacteria	WP_004093409.1	80.078	M	AUG

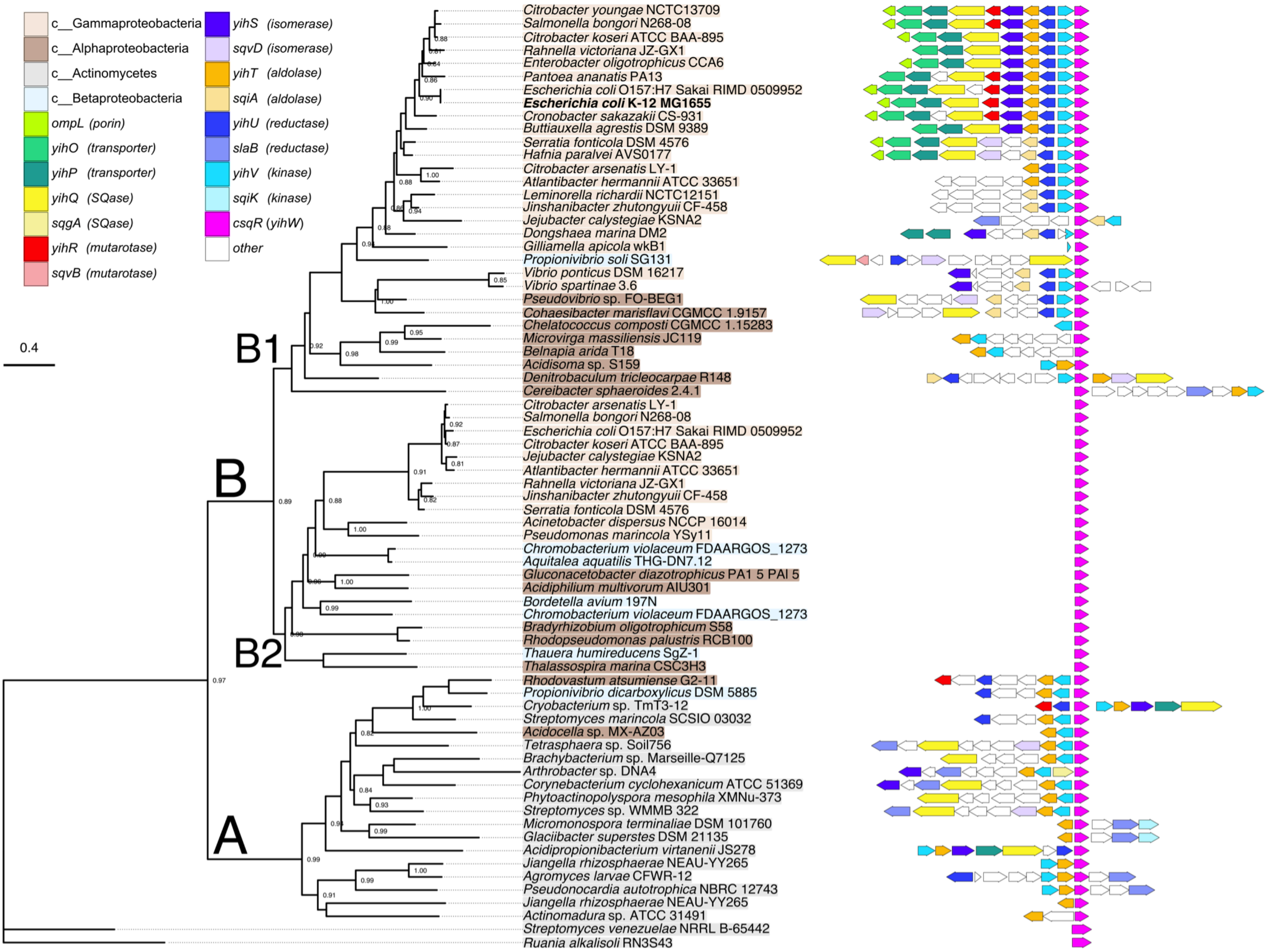

Figure S1. Phylogenetic tree of CsqR (YihW) and its homologs from Gamma-, Beta-, Alpha-, and Actinomycetes. The left panel shows the tree inferred using the maximum likelihood algorithm and rooted manually by the branch leading to the CsqR homologs from *Streptomyces venezuelae* str. NRRL B-65442 and *Ruania alkalisoli* str. RN3S43. The right panel features the genomic neighborhood of the *csqR* (*yihW*) gene in the respective bacteria. Pattern is shown if *csqR* is co-localized with at least one homolog of any other *yih* gene.

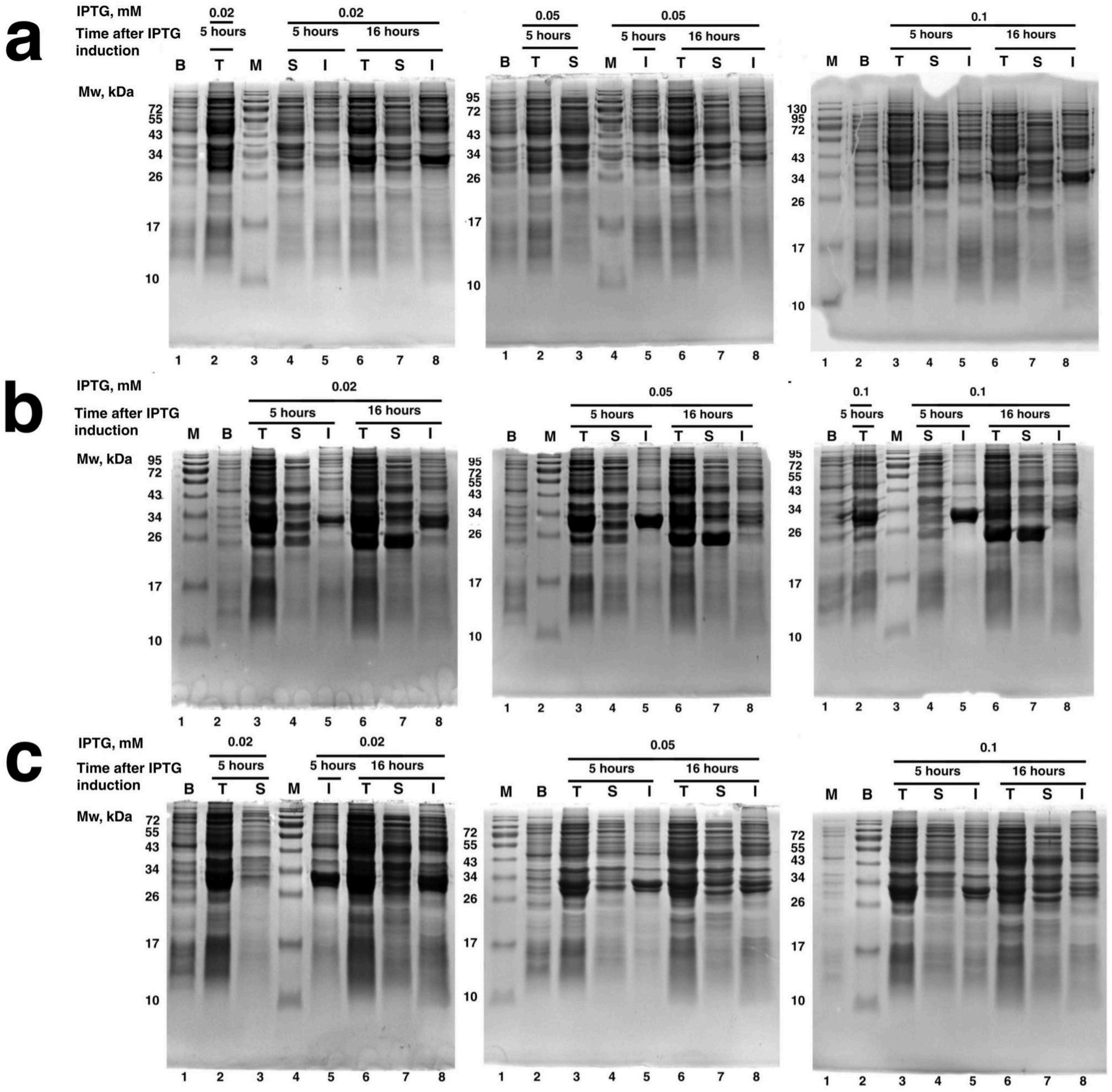

Figure S2. Production of recombinant CsqR protein in *E. coli* OverExpress C41(DE3) **(a)**, *E. coli* BL21-CodonPlus(DE3)-RIL **(b)**, and *E. coli* BL21*(DE3) **(c)** after 5 and 16 hours of growth on the LB medium following IPTG induction (final concentrations 0.02, 0.05 and 0.1 mM). B – before induction, T – total cellular protein fraction, S – soluble fraction, I – insoluble fraction, M – protein molecular weight marker (Prestained Protein Marker #P7712, NEB).

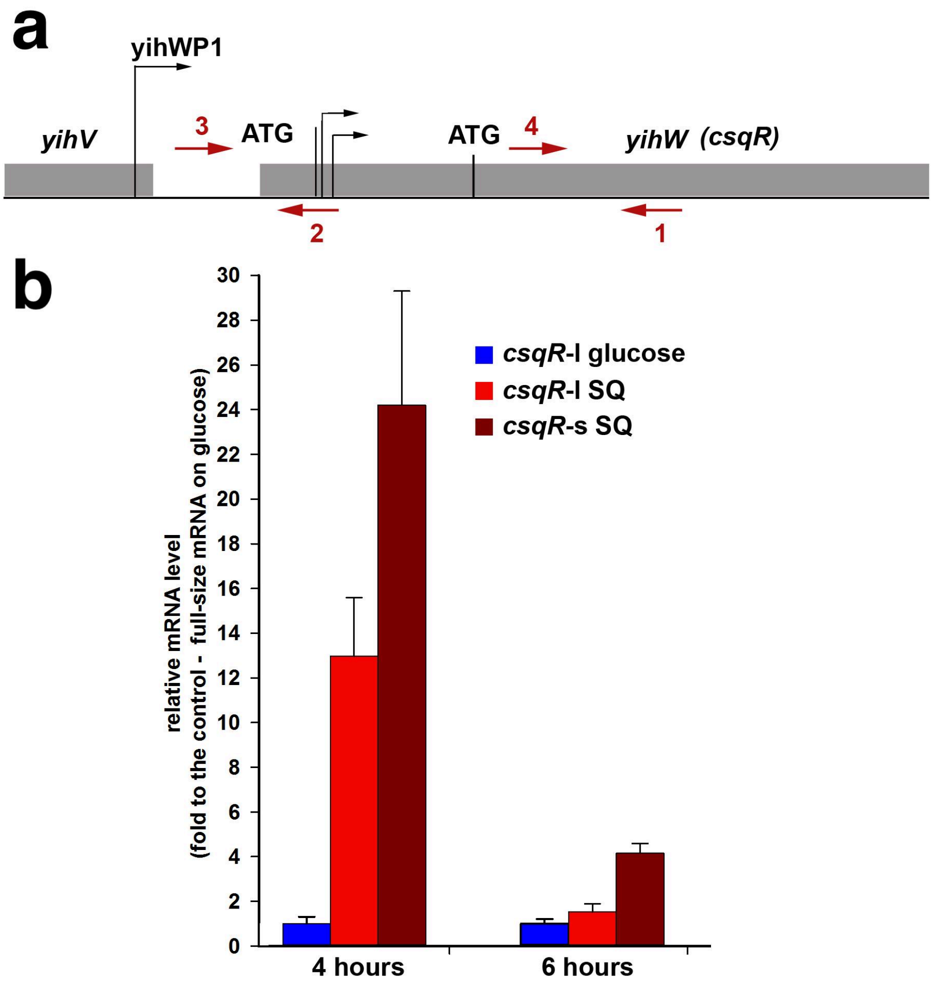

Figure S3. **(a)** Scheme of the *csqR (yihW)* genomic locus and primers used for qRT-PCR. Primers 2+3 were used to detect long form (*csqR*-l), primers 1+4 were used to detect additional expression of shorter mRNA (*csqR*-s) from additional promoters. Promoters were mapped in [12]. **(b)** qRT-PCR data for *csqR*-l- and *csqR*-s-mRNA*,* after 4 and 6 hours of growth on D-glucose or SQ as a sole carbon source. No changes were detected in *hns*-mRNA levels that were used as controls. mRNA levels are expressed relative to the parent strain grown on glucose.

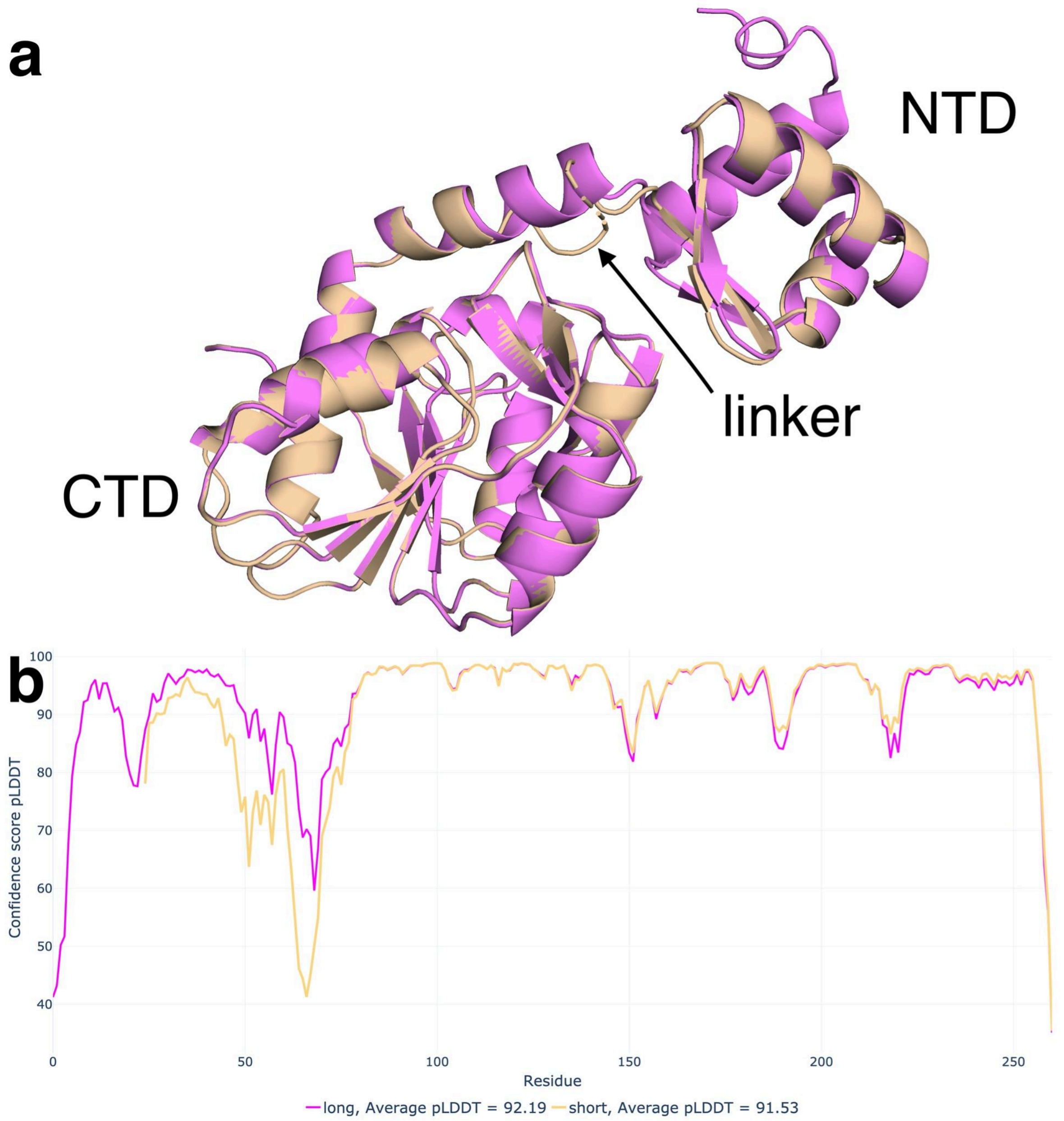

Figure S4. Comparison of the models predicted for CsqR-l and CsqR-s with AlphaFold. (**a**) Flexible structural alignment of CsqR-l (pink) and CsqR-s (beige). (**b**) The AlphaFold per-residue confidence score (pLDDT) predicted for CsqR-l and and CsqR-s.

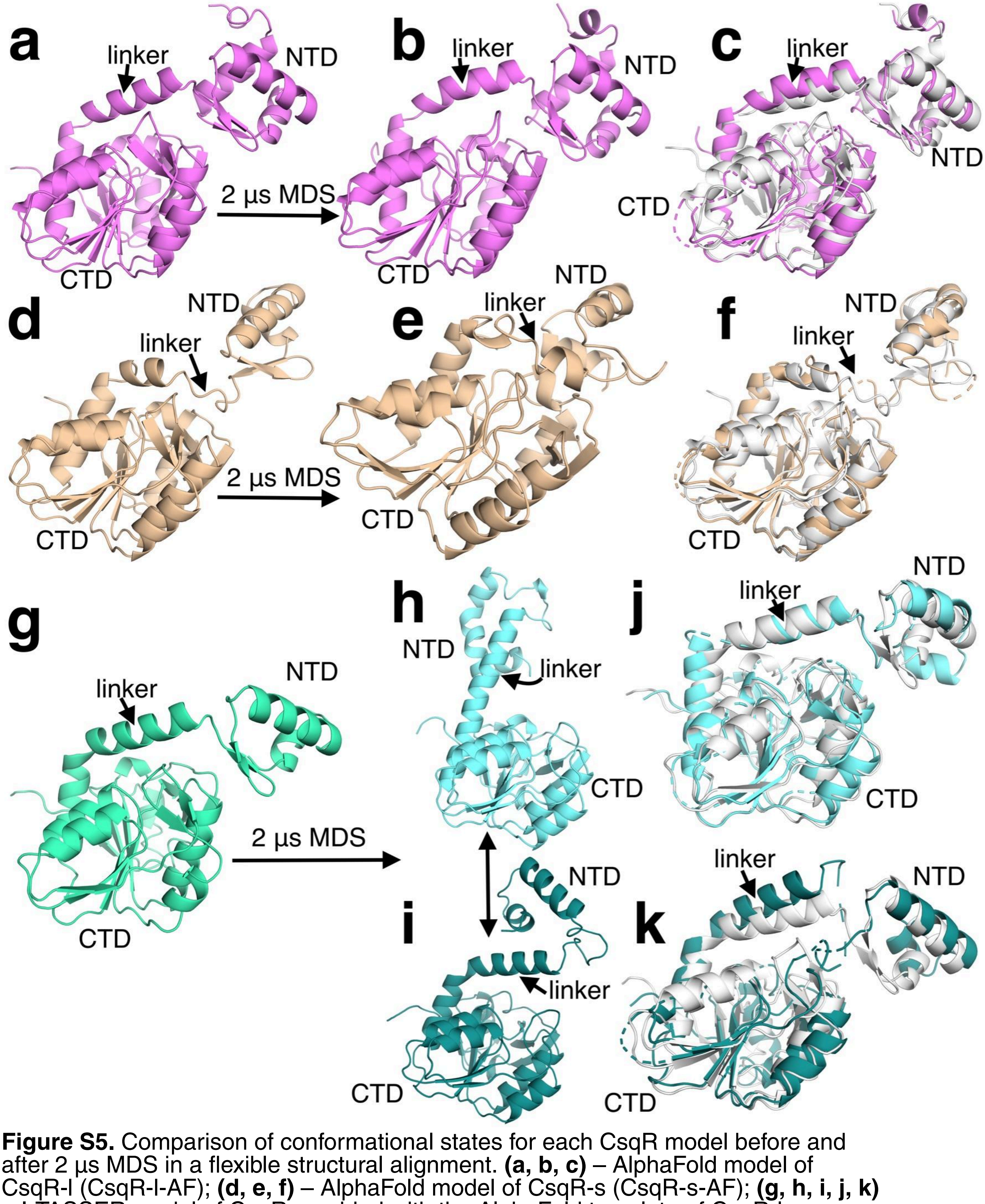

Figure S5. Comparison of conformational states for each CsqR model before and after 2 µs MDS in a flexible structural alignment. **(a, b, c)** – AlphaFold model of CsqR-l (CsqR-l-AF); **(d, e, f)** – AlphaFold model of CsqR-s (CsqR-s-AF); **(g, h, i, j, k)** – I-TASSER model of CsqR-s guided with the AlphaFold template of CsqR-l (CsqR-s-IT). Two main conformations were found for CsqR-s-IT: "open" **(h, j)** and "compact" **(i, k)**. Starting conformations are marked gray on the structural alignments **(c, f, j, k)**.

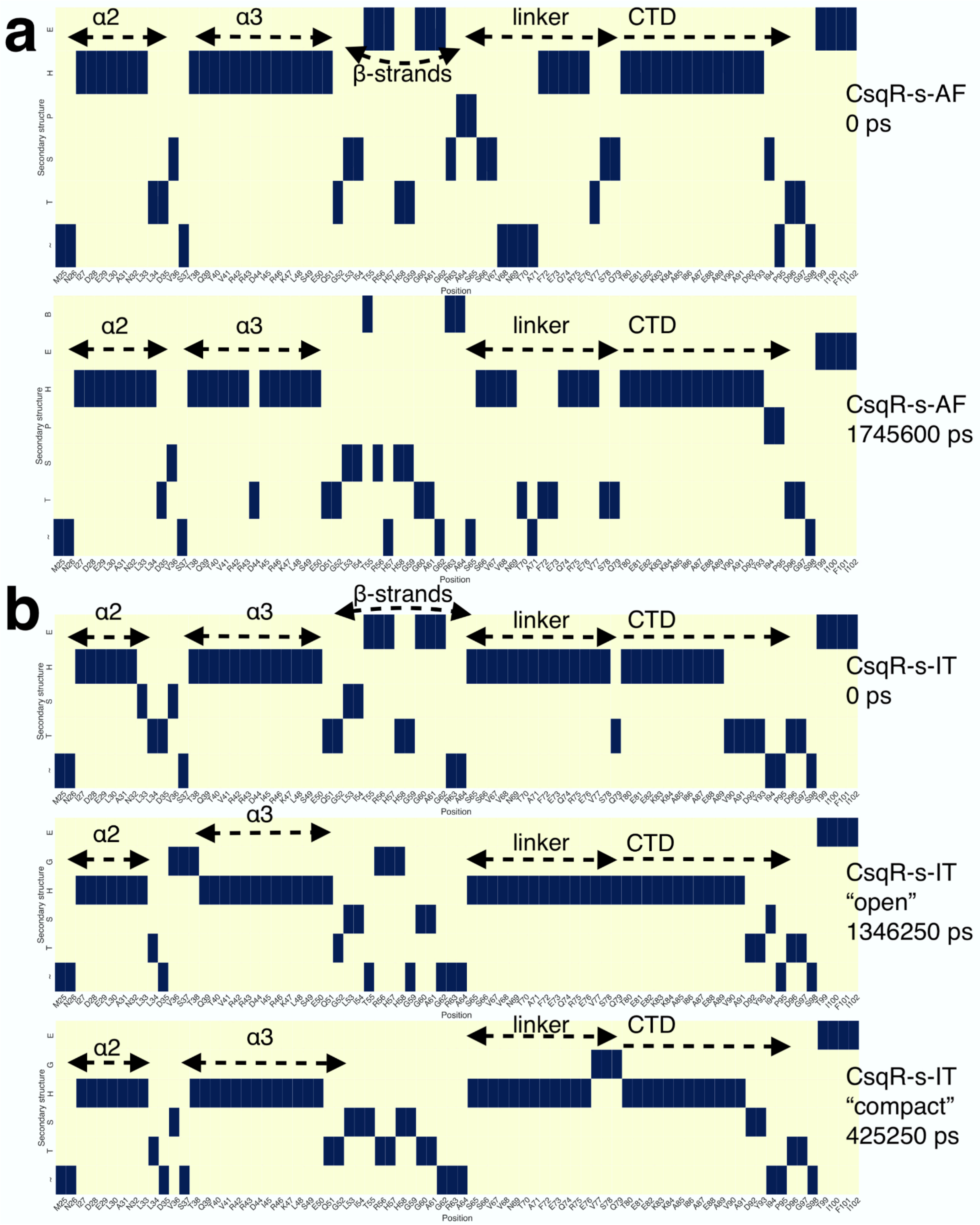

Figure S6. Per-residue assignment of secondary structure obtained by the DSSP algorithm [58] for the CsqR-s models. Amino-acid residues are numbered according to CsqR-l. Positions 25 – 102 are shown. Single character code denotes β-strand-like elements (B – residue in isolated β-bridge; E – extended strand, participates in β ladder), helix- (G – 3_{10}-helix; H – α-helix), and coil-like elements (P – κ-helix (poly-proline II helix); S – bend; T – hydrogen-bonded turn; ~ – loop)). **(a)** AlphaFold model of CsqR-s, before and after MDS. **(b)** I-TASSER model of CsqR-s guided with the AlphaFold template of CsqR-l, before and after MDS. Time of frames for representative conformations is shown (ps). α2 and α3 correspond to α-helices from NTD of CsqR-l.

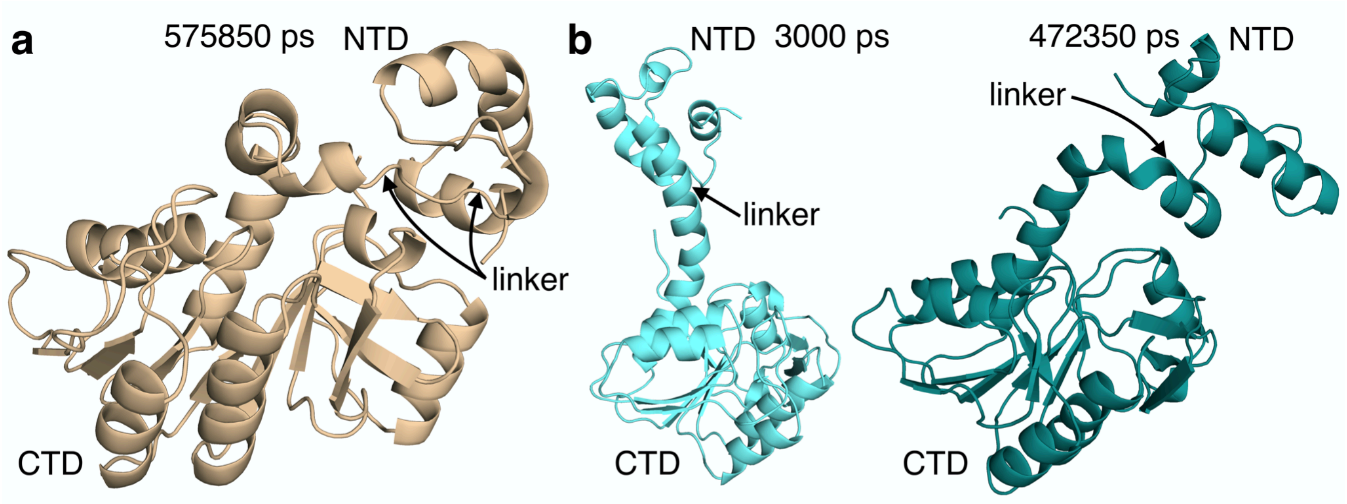

Figure S7. Representative conformations during MDS when temperature of the protein was set to 350 K. **(a)** CsqR-s-AF model; **(b)** CsqR-s-IT model, "open" conformation (frame time of the respective cluster centroid is 3000 ps) and "compact" conformation (frame time of the respective cluster centroid is 472350 ps).

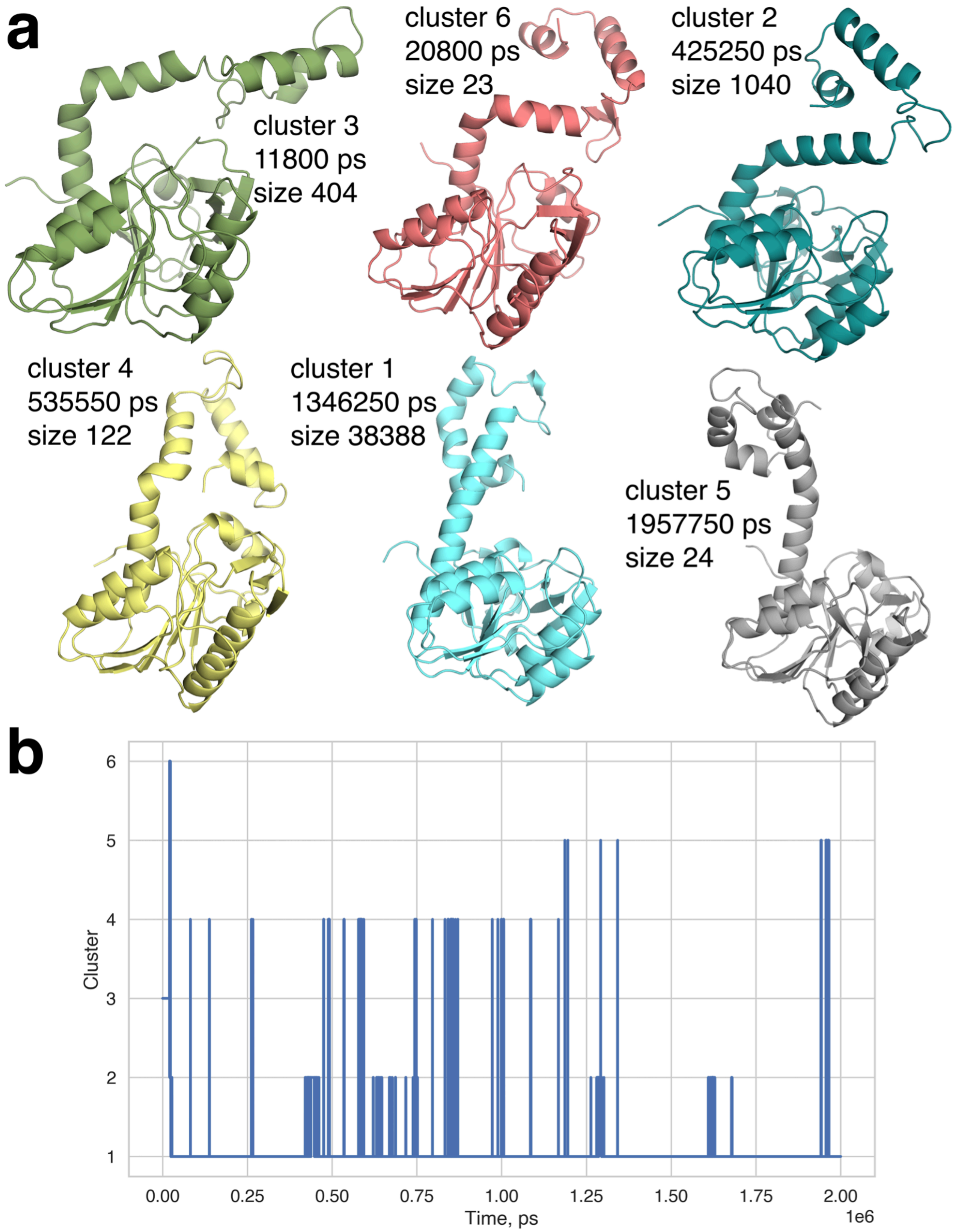

Figure S8. Cluster analysis of the MDS trajectory obtained for the CsqR-s-IT model.
(a) Structures of clusters ordered by the time of the respective centroid frame.
(b) Cluster membership vs simulation time.

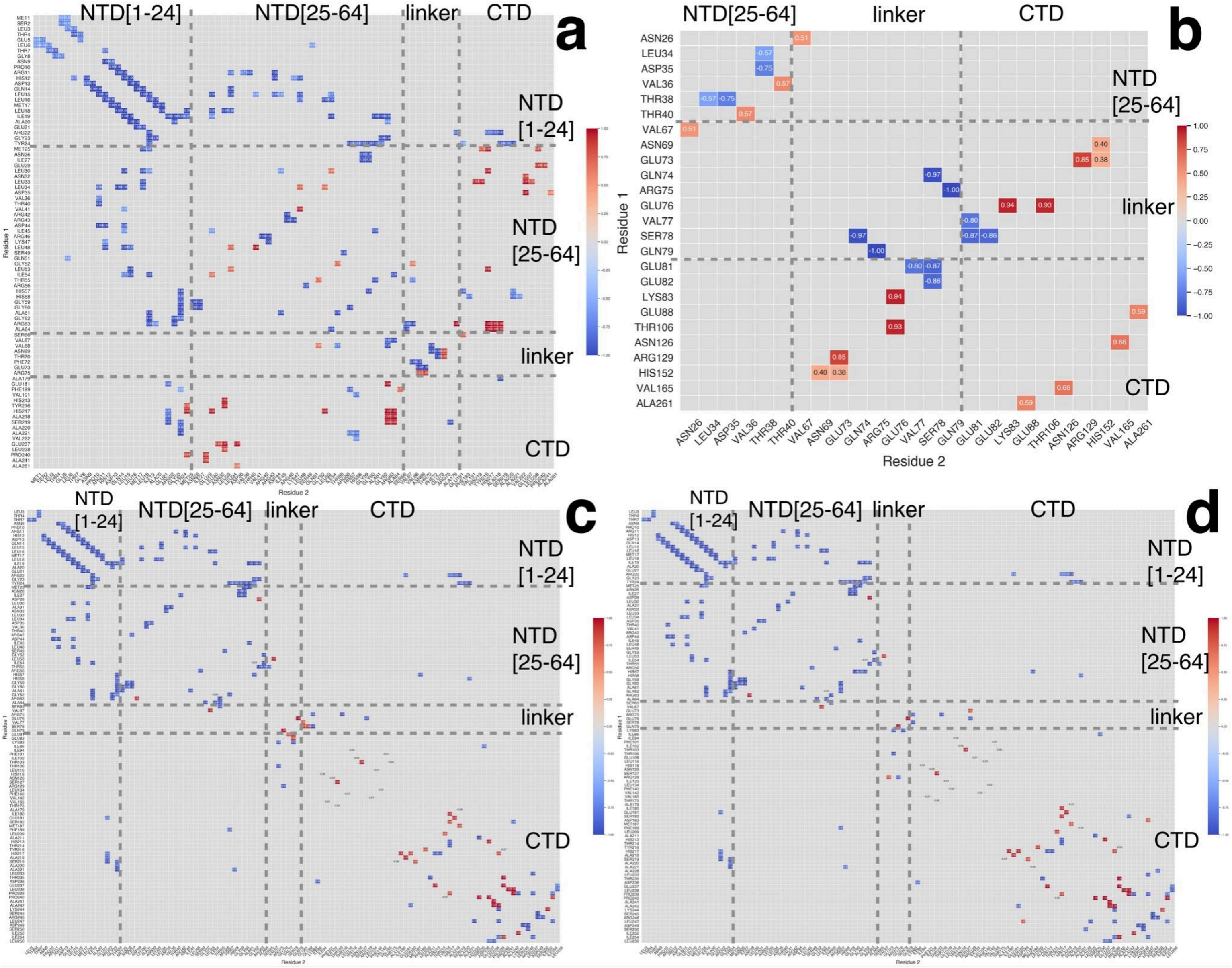

Figure S9. Heatmaps illustrating frequency difference of residue-residue contacts. Changes in frequency of residue-residue contacts in MDS trajectories when switching **(a)** from CsqR-l to CsqR-s-AF; **(b)** from CsqR-s-IT (open) to CsqR-s-IT (compact); **(c)** from CsqR-l to CsqR-s-IT (open); and **(d)** from CsqR-l to CsqR-s-IT (compact). Red color corresponds to emerging contacts (frequency difference > 0), blue color corresponds to disappearing one (frequency difference < 0). Threshold for minimal frequency difference was 0.6 **(a, c, d)** or 0.3 **(b)**. Boundaries of the NTD (residues 1 - 64), the interdomain linker (residues 65 - 79), and the CTD (residue number ≥ 80) were assigned based on secondary structure prediction (see **Figure S6**). Amino-acid residues in CsqR-s are numbered according to CsqR-l.

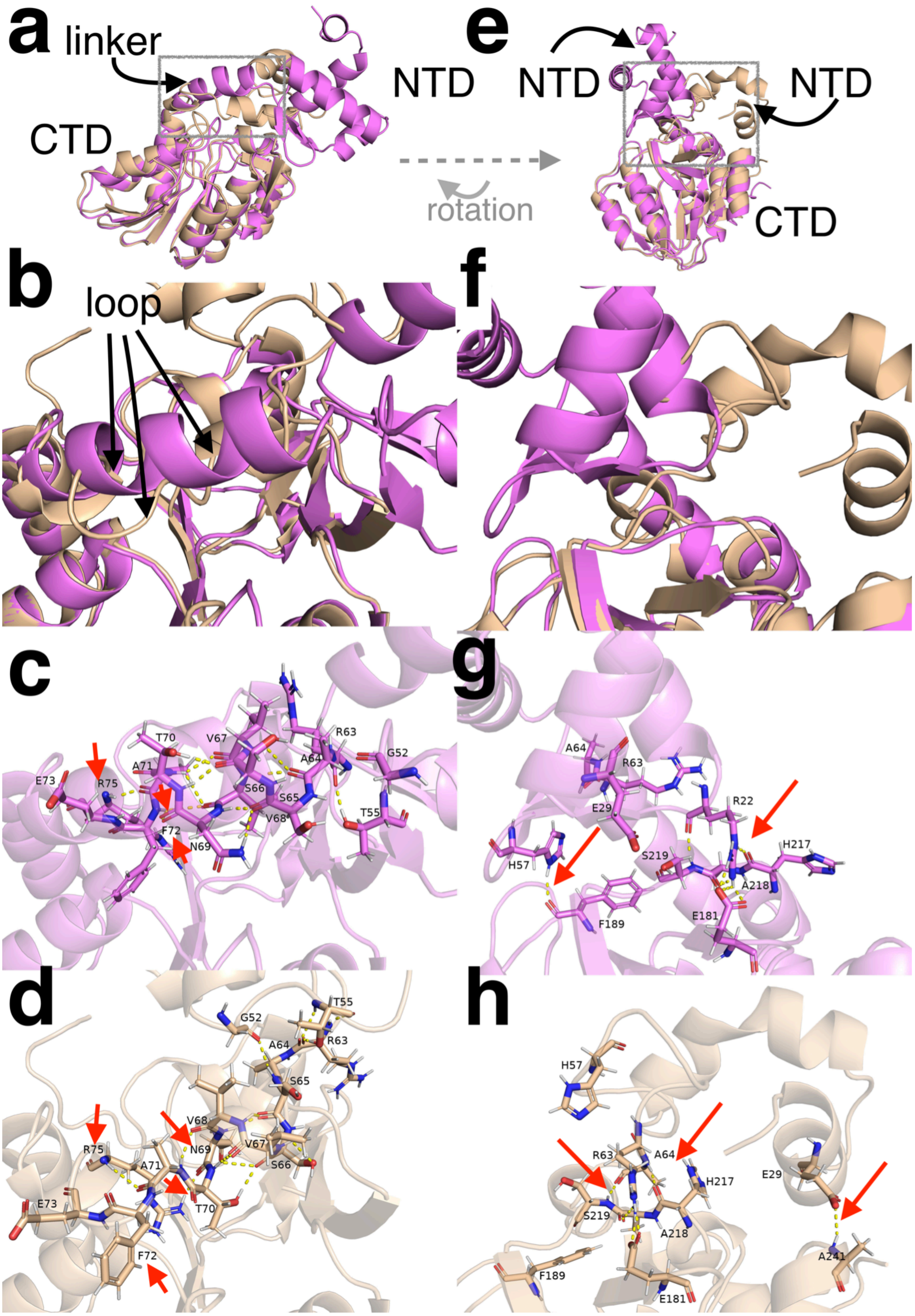
a
linker
NTD
CTD
rotation
e
NTD
NTD
CTD
b
loop
f
c
T70
V67
R63
G52
A64
E73
R75
A71
S66
S65
V68
T55
F72
N69
g
A64
R63
R22
E29
H217
S219
H57
A218
F189
E181
d
T55
G52
A64
R63
S65
V68
R75
N69
V67
A71
S66
T70
E73
F72
h
H57
R63
A64
H217
E29
S219
A218
A241
F189
E181

Figure S10 Residue-residue contacts which frequency significantly differed between CsqR-l-AF and CsqR-s-AF during MDS. Contacts within the interdomain linker **(a, b, c, d)** and contacts between the NTD and the CTD (**e, f, g, h**) are shown. Representative structures of CsqR-l (pink) and CsqR-s-AF (beige) were aligned, and hydrogen bonds (dashed yellow line) of respective residues were obtained using PyMol v2.5.2. For simplicity, only hydrogen bonds related to the contacts from the main text are shown. Red arrows indicate the least common contacts between the two models. **(a, e)** The CsqR-s-AF structure superimposed on the CsqR-l structure; **(b)** the interdomain linker zoomed in after a slight rotation of the **(a)**; **(c)** the interdomain linker of CsqR-l; **(d)** the interdomain linker of CsqR-s-AF; **(f)** the NTD contacting with the CTD zoomed in after a slight rotation of the **(e)**; **(g)** contacts between the NTD and the CTD in CsqR-l; **(h)** contacts between the NTD and the CTD in CsqR-s-AF. Amino-acid residues in CsqR-s are numbered according to CsqR-l.

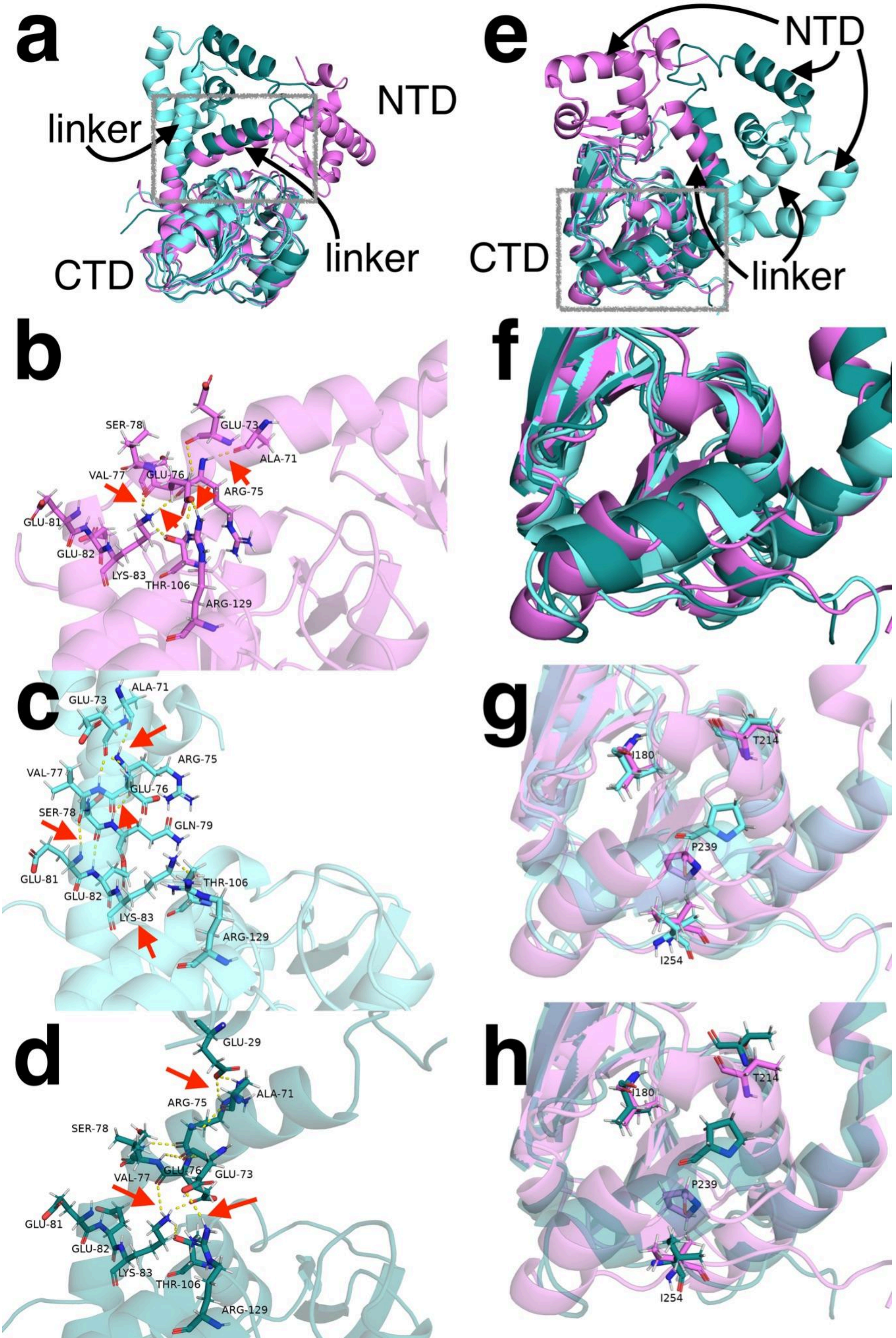

Figure S11. Residue-residue contacts which frequency significantly differed between

CsqR-l-AF and CsqR-s-IT models during MDS. CsqR-l is pink, CsqR-s-IT in the open conformation is light blue, and CsqR-s-IT in the compact conformation is deep teal. Representative structures of CsqR models were aligned, and hydrogen bonds (dashed yellow line) of respective residues were obtained using PyMol v2.5.2. For simplicity, only hydrogen bonds related to the contacts from the main text are shown. Red arrows indicate the least common contacts between the two models. **(a, e)** The CsqR-s-IT models superimposed on the CsqR-l structure; (**b**) the interdomain linker of CsqR-l; **(c)** the interdomain linker of CsqR-s-IT (open); **(d)** the interdomain linker of CsqR-s-IT (compact); **(f)** a region of the CTD that differed by its structural arrangement between CsqR-s-IT and CsqR-l models according to **Figure S9**c-d; **(g)** different structural arrangement of the CTD region in CsqR-s-IT (open) and CsqR-l; **(h)** different structural arrangement of the CTD region in CsqR-s-IT (compact) and CsqR-l. For (**f, g, h)** only four residues are depicted to show positional correspondence between the depicted region and the CTD region from the **Figure S9**c-d. Amino-acid residues in CsqR-s are numbered according to CsqR-l.

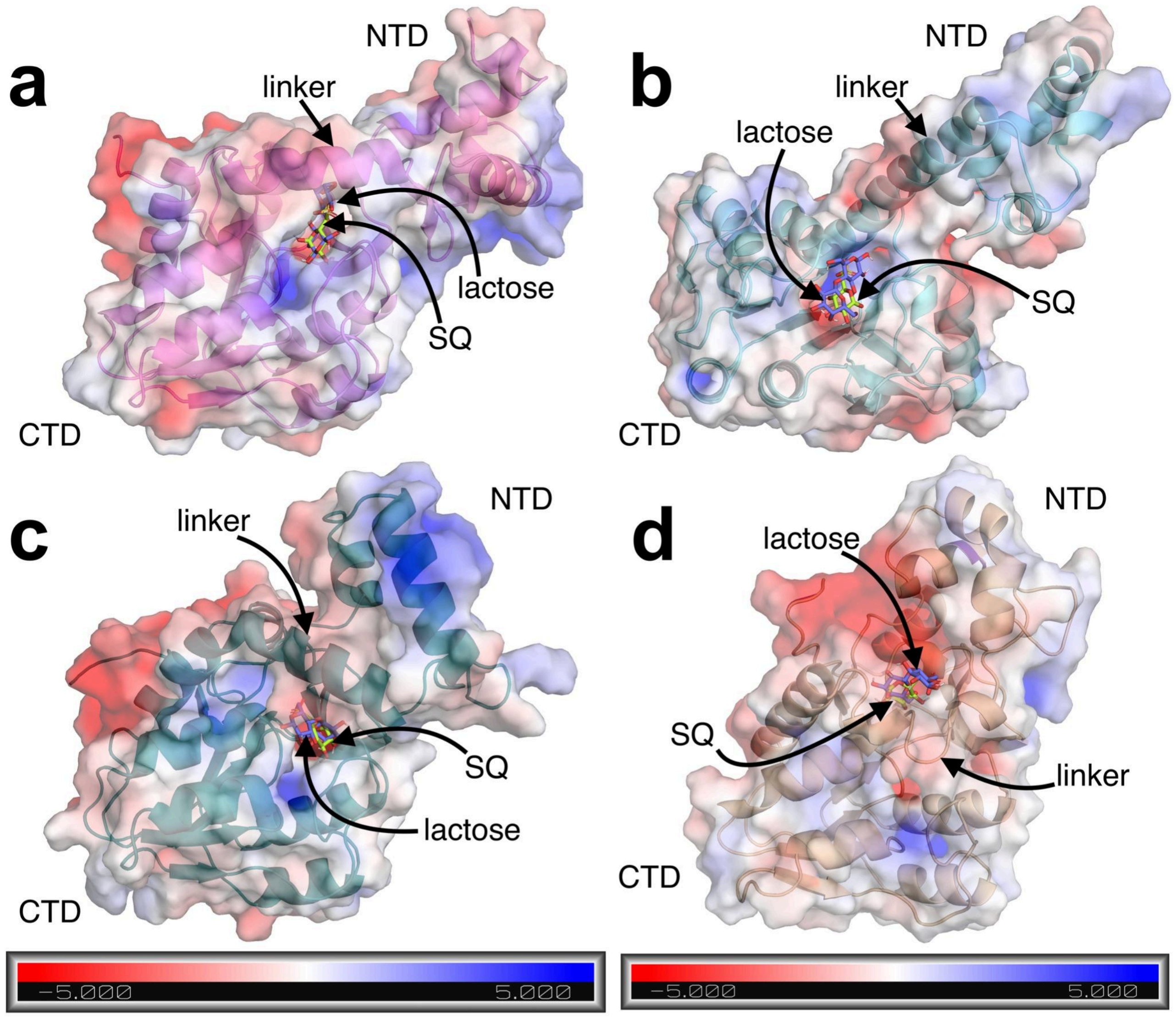

Figure S12. Molecular docking of sulfoquinovose and lactose to CsqR-I **(a)**, CsqR-s-IT in the open conformation **(b)**, CsqR-s-IT in the compact conformation **(c),** and CsqR-s-AF **(d)**. The best binding modes of the ligands are shown. Surfaces are colored according to the electrostatic potential values from negative red to positive blue.

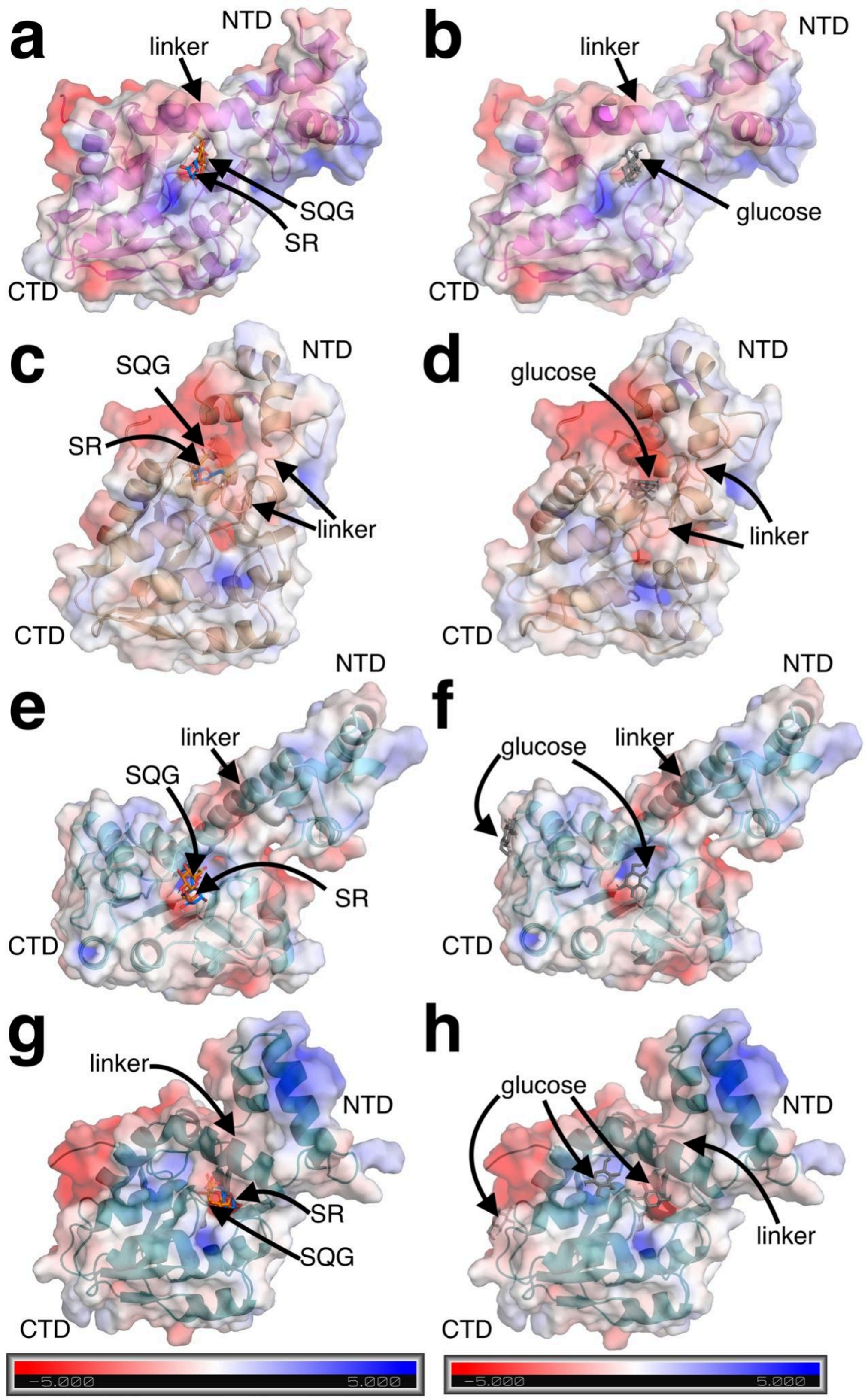

Figure S13. Molecular docking of SR, SQG, and glucose to the CsqR-l and CsqR-s models: **(a, b)** – CsqR-l-AF; **(c, d)** – CsqR-s-AF; **(e, f)** – CsqR-s-IT in the open conformation; **(g, h)** – CsqR-s-IT in the compact conformation. The best binding modes of the ligands are shown. Surfaces are colored according to the electrostatic potential values from negative red to positive blue.

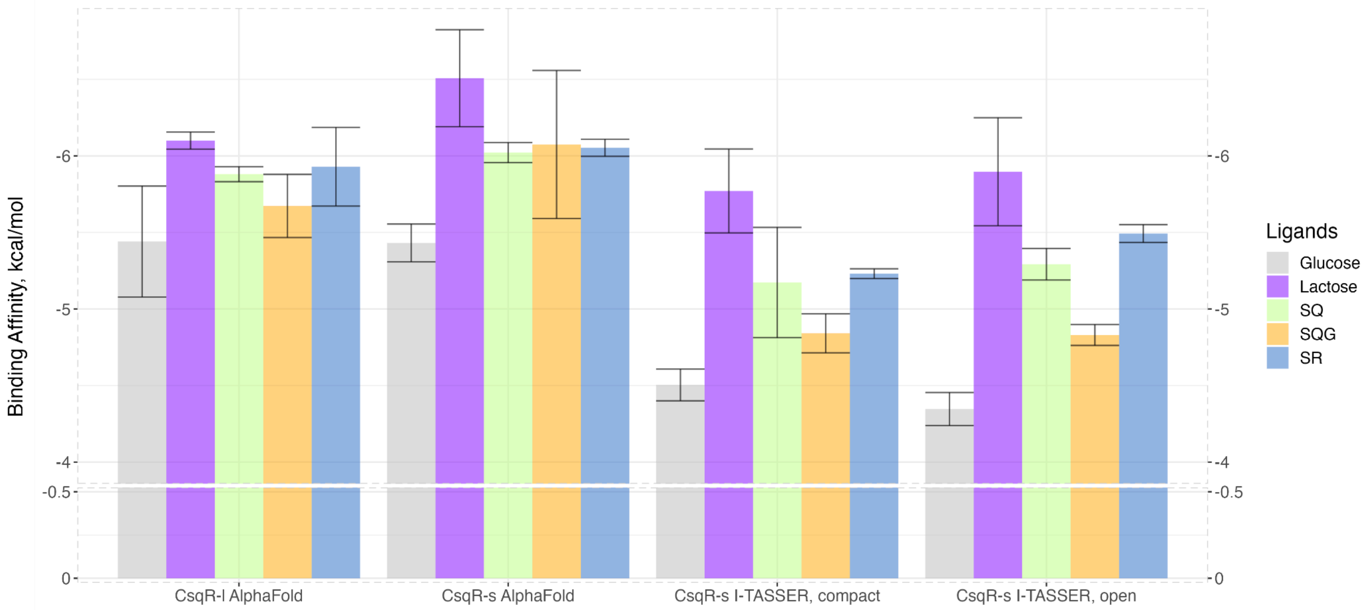

Figure S14. Free Energy of binding (ΔG, kcal/mol) for glucose, lactose, sulfoquinovose, sulfoquinovosyl glycerol, or sulforhamnose, estimated by AutoDock Vina for the models of CsqR-l and CsqR-s based on five computational runs. Error bars represent mean value ± standard deviation.

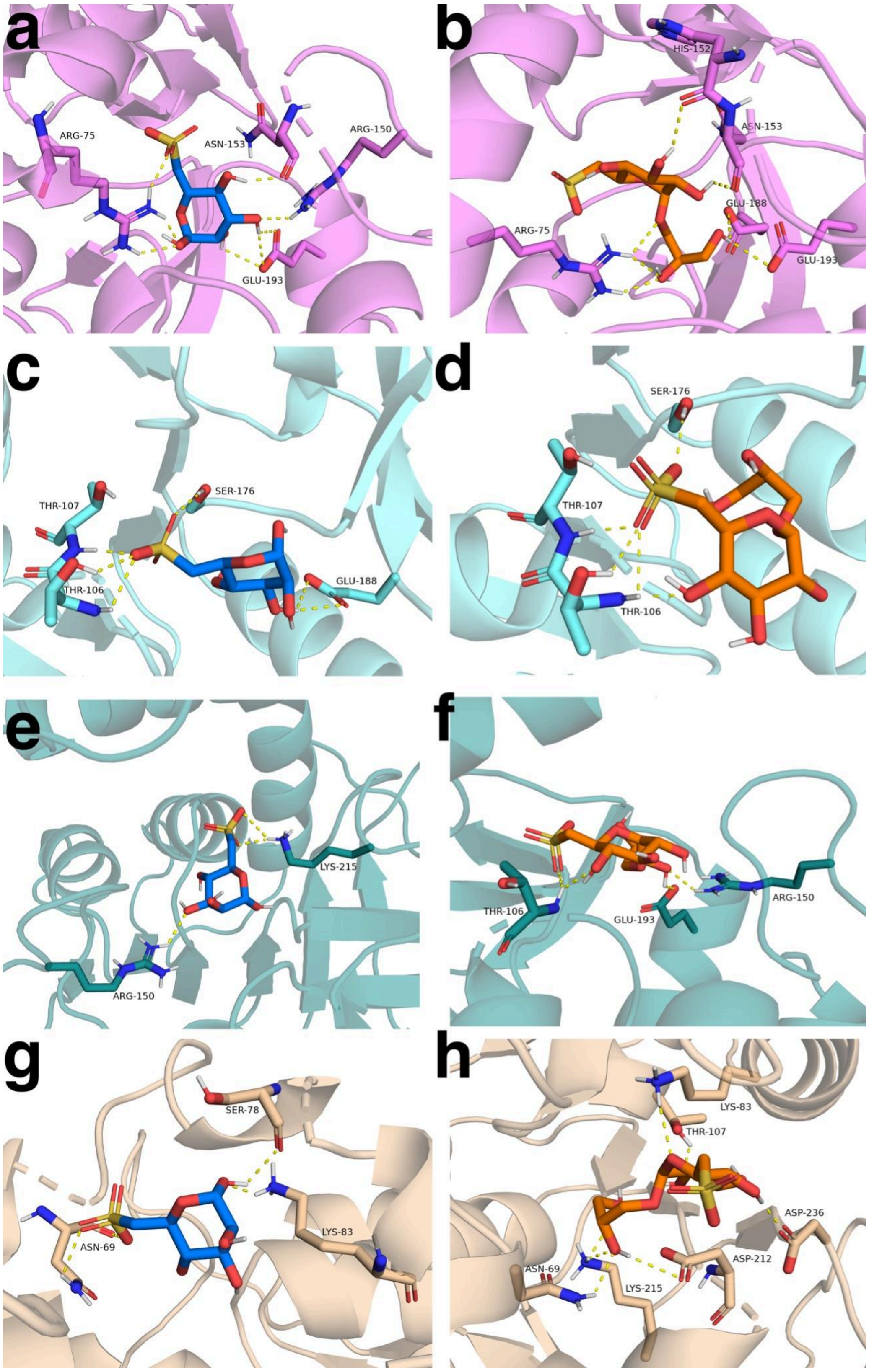

Figure S15. Molecular docking of SR and SQG to CsqR. Ligand binding sites of CsqR models were predicted by AutoDock Vina. Within each protein-ligand complex, the best ligand mode is shown: **(a)** CsqR-l and SR; **(b)** CsqR-l and SQG; **(c)** CsqR-s-IT (open) and SR; **(d)** CsqR-s-IT (open) and SQG; **(e)** CsqR-s-IT (compact) and SR; **(f)** CsqR-s-IT (compact) and SQG; **(g)** CsqR-s-AF and SR; **(h)** CsqR-s-AF and SQG. Polar contacts are marked by a dashed yellow line. Amino-acid residues in CsqR-s are numbered according to CsqR-l.

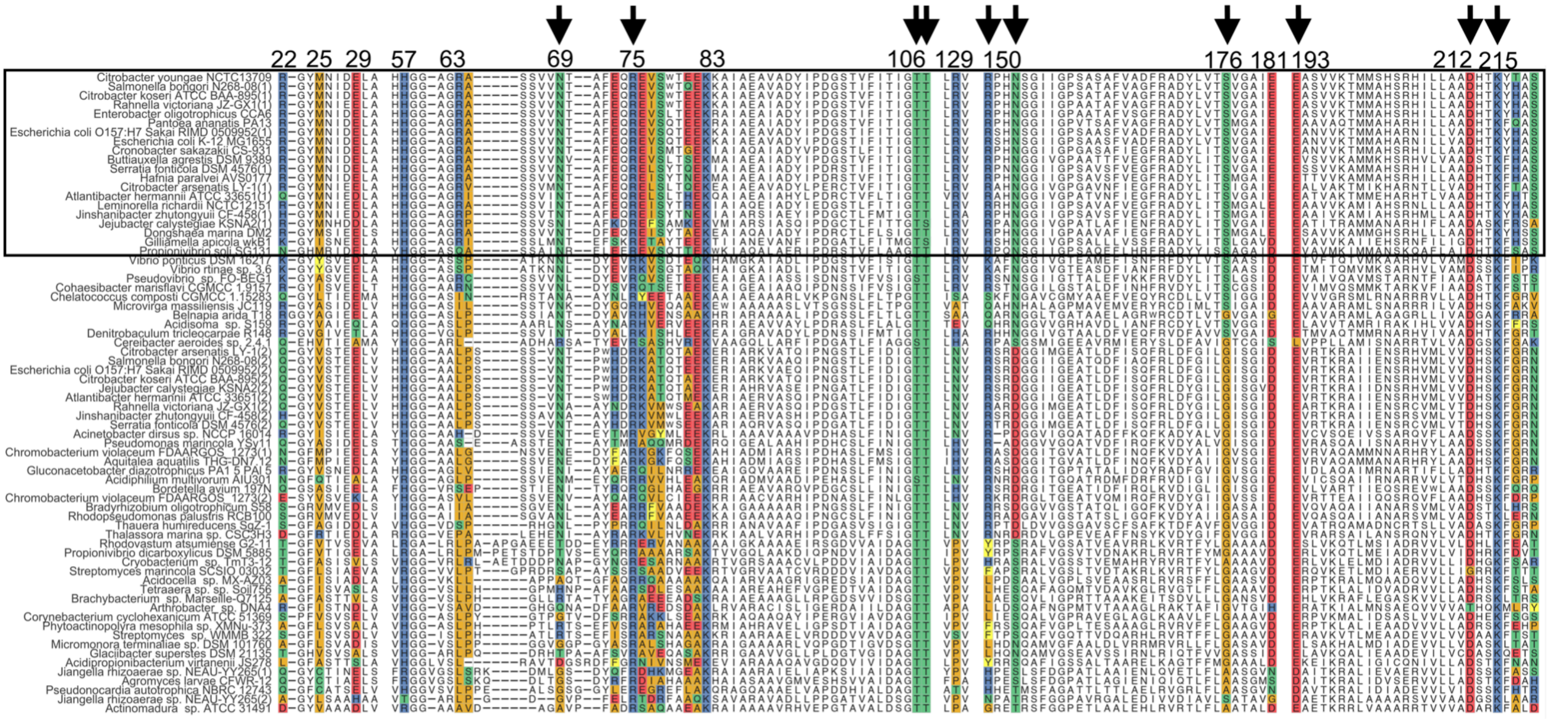

Figure S16. Multiple sequence alignment of CsqR homologs. A part of the alignment (54 – 64, 90 – 148, 171 – 173, 193 – 224, 236 – 262 positions) is shown. CsqR homologs with Met25 are in the black frame. Positions mentioned in the text are highlighted: Arg22, Met25, Glu29, His57, Arg63, Ala64, Asn69, Glu73, Arg75, Glu76, Ser78, Glu81, Glu82, Lys83, Thr106, Thr107, Arg129, Arg150, Asn153, Ser176, Glu181, Glu193, Asp212, Lys215, His217, and Ser219. Numbering of amino acid residues in the CsqR-s is according to CsqR-I. Residues predicted in ligand binding sites are indicated with arrows.

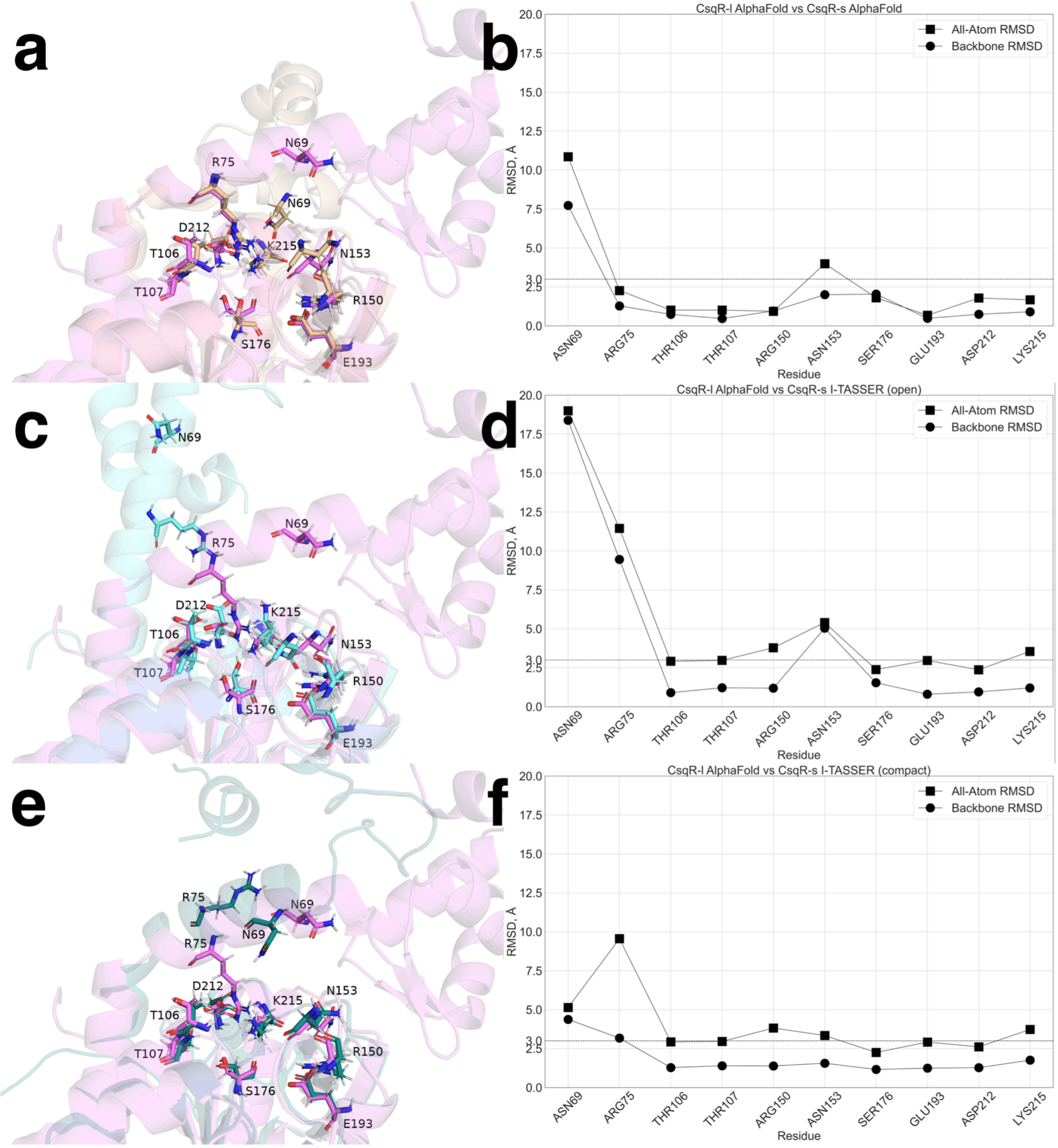

Figure S17. Spatial comparison of residues predicted in a ligand binding site of CsqR. Each CsqR-s model was aligned to CsqR-l. CsqR-l is pink, CsqR-s-AF is beige, CsqR-s-IT in the open conformation is light blue, CsqR-s-IT in the compact conformation is deep teal. Structural alignment and all-atom and backbone RMSD values of the respective residues obtained for: **(a, b)** CsqR-l and CsqR-s-AF; **(c ,d)** CsqR-l and CsqR-s-IT (open); and **(e, f)** CsqR-l and CsqR-s-IT (compact). Amino-acid residues in CsqR-s are numbered according to CsqR-l.

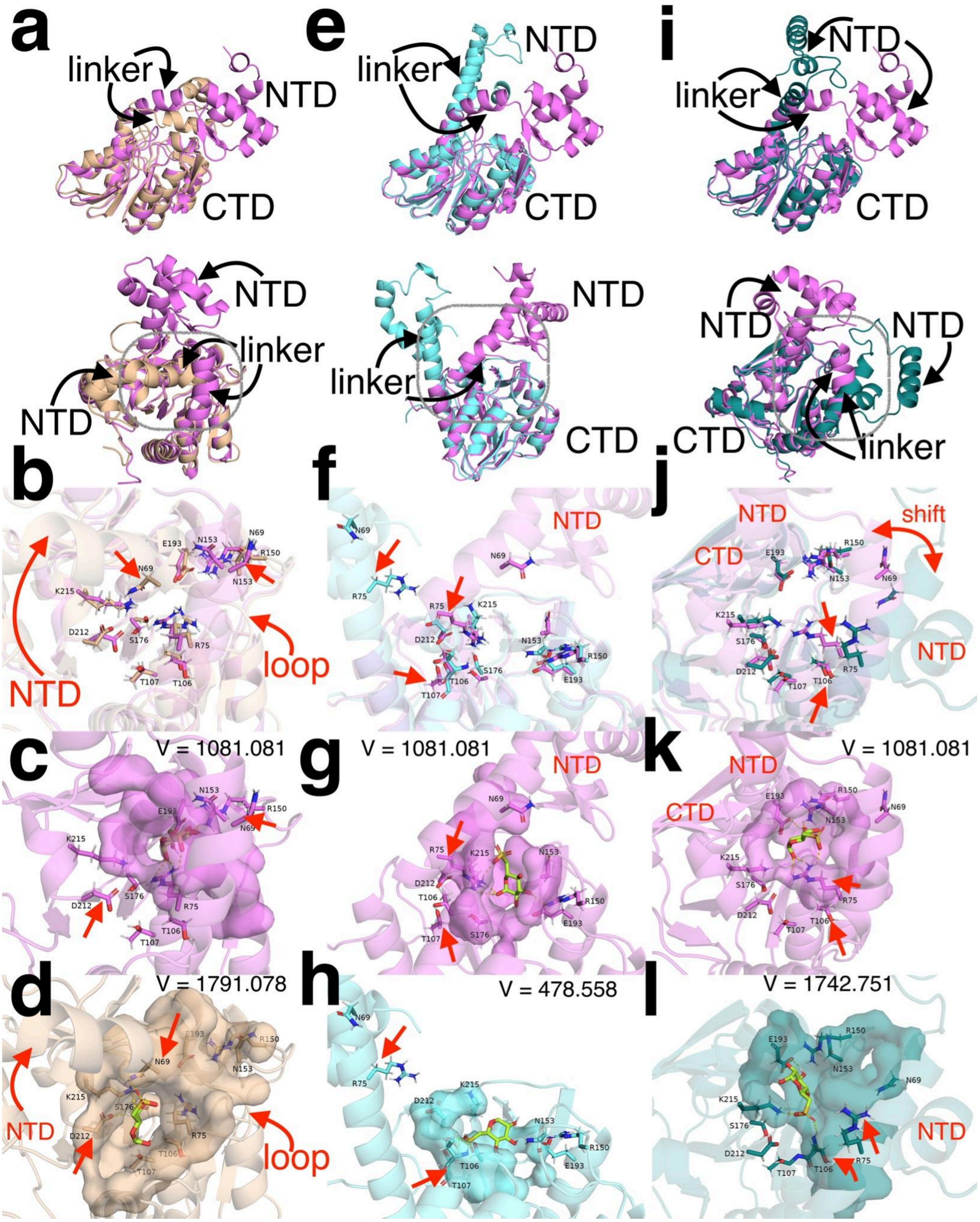

Figure S18. Comparison of putative binding pockets and ligand binding sites between the CsqR-l and CsqR-s models. CsqR-s-IT in the open conformation is light blue, CsqR-s-IT in the compact conformation is deep teal. Red arrows indicate differences in ligand binding sites between the CsqR-s and CsqR-l models. For

simplicity, only polar contacts with SQ are shown for each model. Surfaces represent binding pockets predicted by Fpocket v4.0 [64]. Volume of a binding pocket is provided in Å^3. **(a, e, i)** Structural alignment of CsqR-l and either CsqR-s-AF **(a)**, CsqR-s-IT (open) **(e)**, or CsqR-s-IT (compact) **(i)**. Each structural alignment is provided from two angles. **(b, f, j)** Comparison of a ligand binding site between CsqR-l and either CsqR-s-AF **(b)**, CsqR-s-IT (open) **(f)**, or CsqR-s-IT (compact) **(j).** **(c, d, g, h, k, l)** Predicted binding pocket and ligand binding site of CsqR-l **(c, g, k),** CsqR-s-AF **(d)**, CsqR-s-IT (open) **(h)**, and CsqR-s-IT (compact) **(l)**. Amino-acid residues in CsqR-s are numbered according to CsqR-l.

Table S2. Free Energy of binding (kcal/mol) glucose, lactose, sulforhamnose, sulfoquinovosyl glycerol or sulfoquinovose estimated by AutoDock Vina for the long and short CsqR forms. For each computational run, data on three modes with top affinities is provided.

ligand	CsqR model	run	mode	affinity, kcal/mol	rmsd l.b.	rmsd u.b.
Glu	CsqR-l	1	1	-5.1829	0.0	0.0
			2	-5.1671	1.1276	2.7993
			3	-5.1339	1.0646	3.6381
		2	1	-5.9974	0.0	0.0
			2	-5.2459	1.2942	3.3142
			3	-4.9247	1.6178	2.5373
		3	1	-5.0658	0.0	0.0
			2	-5.0125	1.1275	3.647
			3	-4.6841	1.3295	2.946
		4	1	-5.4284	0.0	0.0
			2	-4.9509	1.3135	3.304
			3	-4.7886	1.4691	3.7956
		5	1	-5.5291	0.0	0.0
			2	-5.2361	1.2005	3.1426
			3	-5.1421	1.2942	2.4301
Lac	CsqR-l	1	1	-6.0858	0.0	0.0
			2	-5.4604	1.552	6.1002
			3	-5.4102	2.382	3.6077
		2	1	-6.1574	0.0	0.0
			2	-5.8121	1.7678	1.9114
			3	-5.4793	1.5271	6.158
		3	1	-6.1553	0.0	0.0
			2	-5.8031	1.7848	6.0941
			3	-5.4696	1.5172	6.1302
		4	1	-6.0296	0.0	0.0
			2	-5.5111	1.4389	6.1106
			3	-5.4687	1.5267	1.7616
		5	1	-6.0707	0.0	0.0
			2	-5.4457	1.5157	6.1365
			3	-5.4209	1.4435	5.9644
		1	1	-5.8598	0.0	0.0
			2	-5.7482	1.5939	2.8113
			3	-5.6221	1.2709	1.2709
		2	1	-5.8464	0.0	0.0
			2	-5.5803	1.533	2.9425
			3	-5.0407	1.4004	1.72

ligand	CsqR model	run	mode	affinity, kcal/mol	rmsd l.b.	rmsd u.b.
SQ	CsqR-I	3	1	-5.8761	0.0	0.0
			2	-5.5321	1.5175	2.7666
			3	-5.0852	2.3408	4.9975
		4	1	-5.8551	0.0	0.0
			2	-5.4937	1.5014	2.8875
			3	-4.9973	2.595	4.9318
		5	1	-5.9648	0.0	0.0
			2	-5.6345	1.4855	2.9304
			3	-4.8253	10.1589	11.6512
SQG	CsqR-I	1	1	-5.7465	0.0	0.0
			2	-5.5552	1.7076	2.3457
			3	-5.4787	2.594	4.8914
		2	1	-5.3067	0.0	0.0
			2	-5.1195	1.5932	1.9672
			3	-4.937	11.1725	12.9001
		3	1	-5.7377	0.0	0.0
			2	-5.1943	1.6764	2.7846
			3	-5.0653	10.4196	12.0183
		4	1	-5.7735	0.0	0.0
			2	-5.3164	10.3312	12.1252
			3	-5.2681	2.5125	5.7498
		5	1	-5.8019	0.0	0.0
			2	-5.2767	1.5533	2.7333
			3	-5.1029	2.557	5.4976
SR	CsqR-I	1	1	-6.1513	0.0	0.0
			2	-5.3548	1.6233	2.8287
			3	-4.9546	2.1597	5.1385
		2	1	-5.4845	0.0	0.0
			2	-5.3162	1.6289	2.293
			3	-5.0729	2.1593	4.9782
		3	1	-6.008	0.0	0.0
			2	-5.066	2.2383	4.9029
			3	-4.8171	10.0553	11.297
		4	1	-6.0147	0.0	0.0
			2	-5.4051	1.443	2.9613
			3	-5.2802	2.3326	5.0697
		5	1	-5.9867	0.0	0.0
			2	-5.3248	1.5482	2.935
			3	-4.8915	1.6931	2.0243
			1	-5.4945	0.0	0.0

ligand	CsqR model	run	mode	affinity, kcal/mol	rmsd l.b.	rmsd u.b.
Glu	CsqR-s-AF	1	2	-5.2213	1.4032	4.6034
			3	-5.1502	1.0833	4.0396
		2	1	-5.4917	0.0	0.0
			2	-5.457	8.7629	10.3672
			3	-5.1053	1.5742	3.132
		3	1	-5.4217	0.0	0.0
			2	-5.2039	1.3615	2.9929
			3	-5.1208	1.1047	3.9828
		4	1	-5.2213	0.0	0.0
			2	-5.1384	1.3243	3.7447
			3	-5.1329	1.5597	4.2012
		5	1	-5.5276	0.0	0.0
			2	-5.2526	8.4708	10.0785
			3	-5.2017	1.171	4.1146
Lac	CsqR-s-AF	1	1	-6.328	0.0	0.0
			2	-6.0703	1.8283	6.1286
			3	-5.9766	2.2232	3.6323
		2	1	-6.3931	0.0	0.0
			2	-6.1922	1.8624	6.3167
			3	-6.0632	1.8626	6.1264
		3	1	-7.0702	0.0	0.0
			2	-6.288	3.5272	6.2785
			3	-6.081	2.6413	7.7028
		4	1	-6.412	0.0	0.0
			2	-6.3886	2.4992	3.3534
			3	-6.1778	1.606	5.735
		5	1	-6.3337	0.0	0.0
			2	-6.1915	1.7885	6.0581
			3	-6.0266	1.7269	2.5203
SQ	CsqR-s-AF	1	1	-6.0894	0.0	0.0
			2	-5.9659	1.2972	2.8549
			3	-5.8021	2.3206	2.925
		2	1	-5.9679	0.0	0.0
			2	-5.7639	1.9813	3.3889
			3	-5.3983	1.4501	1.9585
		3	1	-6.0776	0.0	0.0
			2	-6.0051	1.294	2.8876
			3	-5.8612	2.2637	2.782
		4	1	-6.032	0.0	0.0
			2	-5.9759	1.3165	2.7133

ligand	CsqR model	run	mode	affinity, kcal/mol	rmsd l.b.	rmsd u.b.
			3	-5.8084	2.2495	2.8305
		5	1	-5.9419	0.0	0.0
			2	-5.8764	1.2081	2.6727
			3	-5.811	1.9884	3.3255
SQG	CsqR-s-AF	1	1	-5.897	0.0	0.0
			2	-5.8759	1.6204	2.4659
			3	-5.7264	2.2938	4.8785
		2	1	-5.912	0.0	0.0
			2	-5.5344	2.558	5.2006
			3	-5.5196	2.3369	5.9806
		3	1	-5.7108	0.0	0.0
			2	-5.6849	2.2612	4.5741
			3	-5.5788	2.533	5.5659
		4	1	-5.9299	0.0	0.0
			2	-5.5266	1.9756	4.3844
			3	-5.4582	2.3517	6.0461
		5	1	-6.9249	0.0	0.0
			2	-6.463	1.475	2.3511
			3	-5.7351	2.7016	6.2982
SR	CsqR-s-AF	1	1	-6.0495	0.0	0.0
			2	-6.01	0.1526	1.1141
			3	-5.121	1.443	2.1349
		2	1	-6.0706	0.0	0.0
			2	-6.0519	0.1631	1.1332
			3	-6.0358	0.0838	1.1148
		3	1	-6.1119	0.0	0.0
			2	-6.0624	0.1067	1.0894
			3	-5.2222	2.1115	4.8988
		4	1	-5.9617	0.0	0.0
			2	-5.5146	0.9555	1.0833
			3	-5.3752	0.4503	1.169
		5	1	-6.073	0.0	0.0
			2	-6.0543	0.1166	1.1186
			3	-5.2322	2.1248	4.8907
Glu	CsqR-s-IT, compact	1	1	-4.5869	0.0	0.0
			2	-4.4766	6.9675	8.0694
			3	-4.3196	12.9755	14.911
		2	1	-4.4183	0.0	0.0
			2	-4.3955	7.2179	9.6009
			3	-4.3289	25.4561	27.5164

ligand	CsqR model	run	mode	affinity, kcal/mol	rmsd l.b.	rmsd u.b.
		3	1	-4.392	0.0	0.0
			2	-4.3677	7.2612	9.5908
			3	-4.2932	25.3967	27.4669
		4	1	-4.6306	0.0	0.0
			2	-4.5872	27.185	28.7551
			3	-4.4034	20.6287	22.5273
		5	1	-4.4919	0.0	0.0
			2	-4.4668	7.3615	9.719
			3	-4.3036	12.3157	14.2324
Lac	CsqR-s-IT, compact	1	1	-5.5625	0.0	0.0
			2	-5.4952	19.2785	22.1267
			3	-5.3517	1.6091	6.2067
		2	1	-5.6898	0.0	0.0
			2	-5.6286	1.9198	3.402
			3	-5.3351	9.6844	11.2885
		3	1	-6.2456	0.0	0.0
			2	-5.7588	1.7444	3.5548
			3	-5.4923	2.041	5.9926
		4	1	-5.7434	0.0	0.0
			2	-5.6931	1.9716	3.3689
			3	-5.6467	1.8754	6.3847
		5	1	-5.6144	0.0	0.0
			2	-5.234	9.5987	11.1972
			3	-5.1448	1.7102	6.7884
SQ	CsqR-s-IT, compact	1	1	-5.5298	0.0	0.0
			2	-5.2908	2.0923	2.6623
			3	-5.0019	1.6471	2.5492
		2	1	-5.0903	0.0	0.0
			2	-4.7237	11.4406	12.676
			3	-4.7075	11.2632	13.5215
		3	1	-5.525	0.0	0.0
			2	-5.2665	2.0925	2.7323
			3	-4.95	11.4952	13.7095
		4	1	-4.6812	0.0	0.0
			2	-4.6559	15.1194	16.4119
			3	-4.6361	13.624	15.3055
		5	1	-5.0406	0.0	0.0
			2	-4.9498	2.1036	2.7661
			3	-4.8876	1.8407	2.6891

ligand	CsqR model	run	mode	affinity, kcal/mol	rmsd l.b.	rmsd u.b.
SQG	CsqR-s-IT, compact	1	1	-4.8573	0.0	0.0
			2	-4.8007	9.7702	12.0435
			3	-4.792	2.4972	6.2624
		2	1	-4.6869	0.0	0.0
			2	-4.3093	9.4074	12.5257
			3	-4.3084	20.8173	23.2833
		3	1	-5.0388	0.0	0.0
			2	-4.7641	3.0379	6.5404
			3	-4.6468	12.0639	14.9633
		4	1	-4.8178	0.0	0.0
			2	-4.5961	1.6895	2.4679
			3	-4.5559	9.9702	12.6893
		5	1	-4.8053	0.0	0.0
			2	-4.773	2.4497	5.1299
			3	-4.5995	9.2327	11.6858
SR	CsqR-s-IT, compact	1	1	-5.2593	0.0	0.0
			2	-5.1473	2.1298	4.9037
			3	-5.0824	2.1684	5.0715
		2	1	-5.1776	0.0	0.0
			2	-4.9423	2.1673	5.0581
			3	-4.8938	12.4767	13.7746
		3	1	-5.2336	0.0	0.0
			2	-5.1765	2.14	4.8521
			3	-5.0843	2.1877	5.0817
		4	1	-5.2339	0.0	0.0
			2	-5.0549	2.2098	5.0881
			3	-4.9754	11.9581	13.2313
		5	1	-5.2503	0.0	0.0
			2	-5.0328	2.1721	5.0729
			3	-4.8665	2.5282	4.905
Glu	CsqR-s-IT, open	1	1	-4.2546	0.0	0.0
			2	-4.0831	25.0264	25.7564
			3	-4.0032	25.1001	25.867
		2	1	-4.4933	0.0	0.0
			2	-4.4676	25.1715	26.0419
			3	-4.2698	25.3407	26.0968
		3	1	-4.3505	0.0	0.0
			2	-4.1813	25.2775	25.9455
			3	-4.0865	24.9918	25.7211

ligand	CsqR model	run	mode	affinity, kcal/mol	rmsd l.b.	rmsd u.b.
		4	1	-4.2307	0.0	0.0
			2	-4.1464	25.1783	25.7803
			3	-3.9829	25.71	26.3613
		5	1	-4.4037	0.0	0.0
			2	-4.371	25.1451	25.8682
			3	-4.1474	2.5589	4.9163
Lac	CsqR-s-IT, open	1	1	-5.2938	0.0	0.0
			2	-5.2096	2.3296	4.0113
			3	-5.169	1.922	6.0813
		2	1	-5.7084	0.0	0.0
			2	-5.5707	1.2043	6.6665
			3	-5.4041	2.0989	6.3251
		3	1	-5.8451	0.0	0.0
			2	-5.6572	1.7474	6.8444
			3	-5.5364	2.2003	3.2888
		4	1	-6.0194	0.0	0.0
			2	-5.615	1.6856	2.1487
			3	-5.5979	2.1053	6.1331
		5	1	-6.3321	0.0	0.0
			2	-6.2563	1.4224	6.8388
			3	-5.7825	2.1976	3.221
SQ	CsqR-s-IT, open	1	1	-5.2807	0.0	0.0
			2	-5.2457	1.6464	2.3044
			3	-4.9771	2.1194	5.2838
		2	1	-5.2358	0.0	0.0
			2	-5.2223	0.0874	1.0949
			3	-5.1711	1.7084	2.2451
		3	1	-5.2649	0.0	0.0
			2	-5.1785	1.2742	1.9397
			3	-5.1189	1.6506	2.3707
		4	1	-5.4707	0.0	0.0
			2	-5.2743	1.0673	1.3907
			3	-5.1258	1.5792	2.3824
		5	1	-5.211	0.0	0.0
			2	-5.1295	1.6543	2.0776
			3	-5.1116	1.2776	1.9793
SQG	CsqR-s-IT, open	1	1	-4.8777	0.0	0.0
			2	-4.8695	2.5946	5.4776
			3	-4.7344	2.8727	5.2486
			1	-4.7498	0.0	0.0

ligand	CsqR model	run	mode	affinity, kcal/mol	rmsd l.b.	rmsd u.b.
		2	2	-4.6871	2.7587	3.9769
			3	-4.6123	2.3355	5.6383
		3	1	-4.7309	0.0	0.0
			2	-4.6303	2.7431	6.0279
			3	-4.4977	2.3215	3.4219
		4	1	-4.8021	0.0	0.0
			2	-4.7933	2.1613	4.2921
			3	-4.7906	2.1819	5.1024
		5	1	-4.8755	0.0	0.0
			2	-4.8711	2.5625	5.4795
			3	-4.7848	2.7022	3.9555
SR	CsqR-s-IT, open	1	1	-5.4844	0.0	0.0
			2	-5.3854	1.6496	2.5497
			3	-5.2648	1.5473	2.2577
		2	1	-5.5927	0.0	0.0
			2	-5.3069	1.63	2.5045
			3	-4.9474	1.6252	2.4739
		3	1	-5.4819	0.0	0.0
			2	-5.4709	1.7148	2.5744
			3	-5.4246	1.5631	2.3864
		4	1	-5.4521	0.0	0.0
			2	-5.3587	1.6953	2.5528
			3	-5.1748	2.0863	2.9054
		5	1	-5.4529	0.0	0.0
			2	-5.2589	1.7086	2.5827
			3	-4.8489	1.1617	1.809
