# Supplementary material for: Phylogeny and structural modeling of the transcription factor CsqR (YihW) from Escherichia coli
Source: Sci Rep. 2024 Apr 3;14:7852. doi: 10.1038/s41598-024-58492-y (PMC10991401; doi:10.1038/s41598-024-58492-y)

Phylogeny and structural modeling of the transcription factor CsqR (YihW) from *Escherichia coli*

Anna A. Rybina[1*], Roman A. Glushak[2], Tatiana A. Bessonova[3], Artemiy I. Dakhnovets[1], Alexander Y. Rudenko[4], Ratislav M. Ozhiganov[4], Anna D. Kaznadzey[5], Maria N. Tutukina[1,3,5], Mikhail S. Gelfand[1, 5]

[1]Skolkovo Institute of Science and Technology, Moscow, 121205, Russia

[2]Faculty of Biology, Lomonosov Moscow State University, Moscow, 119991, Russia

[3]Institute of Cell Biophysics RAS (Federal Research Center “Pushchino Scientific Center for Biological Research of the RAS”), Pushchino, 142290, Russia

[4]Belozersky Institute of Physico-Chemical Biology, Lomonosov Moscow State University, Moscow, 119991, Russia

[5]Institute for Information Transmission Problems RAS, Moscow, 127051, Russia

*rybinaann@gmail.com

Original uncropped photos of the protein gels made on the iBright 750 (Thermo Scientific)

Fig. S2 a

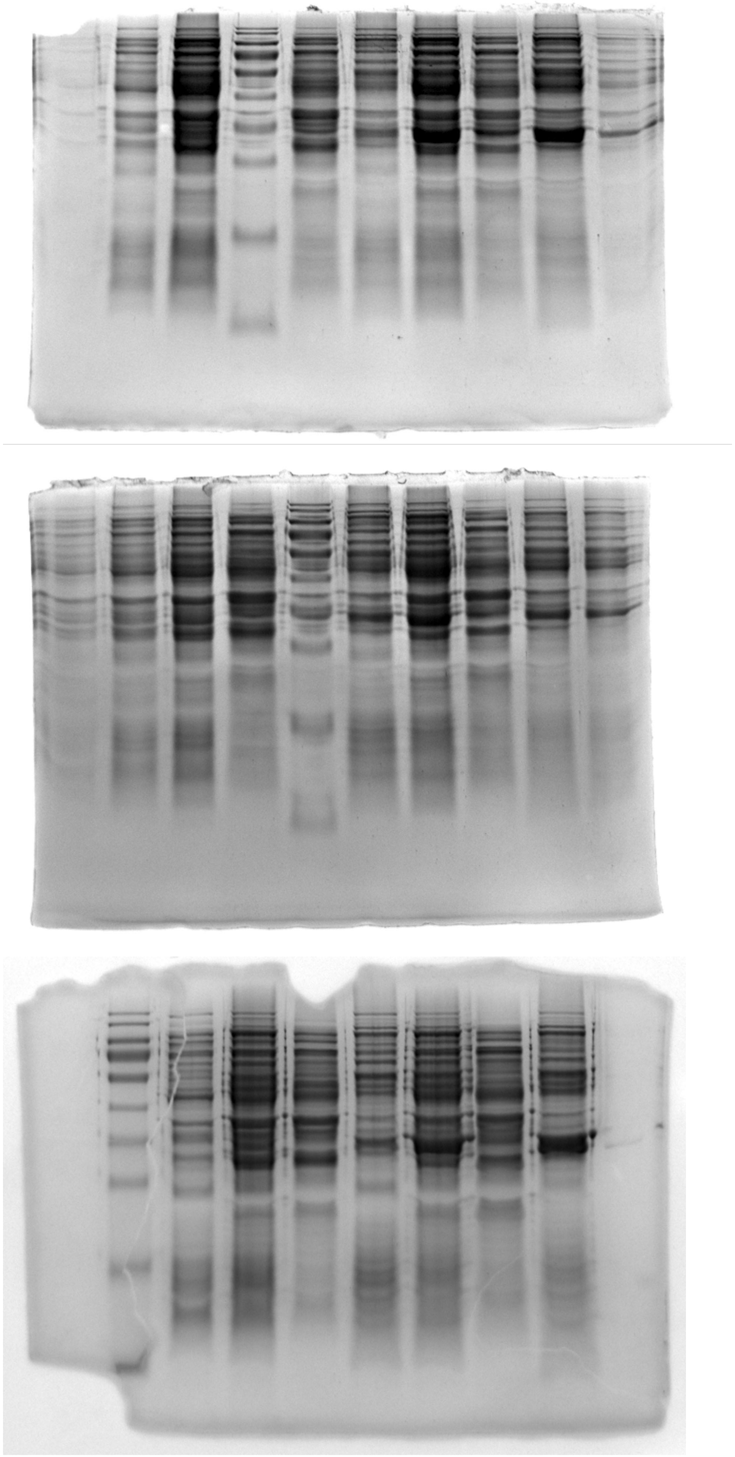

Fig. S2 b

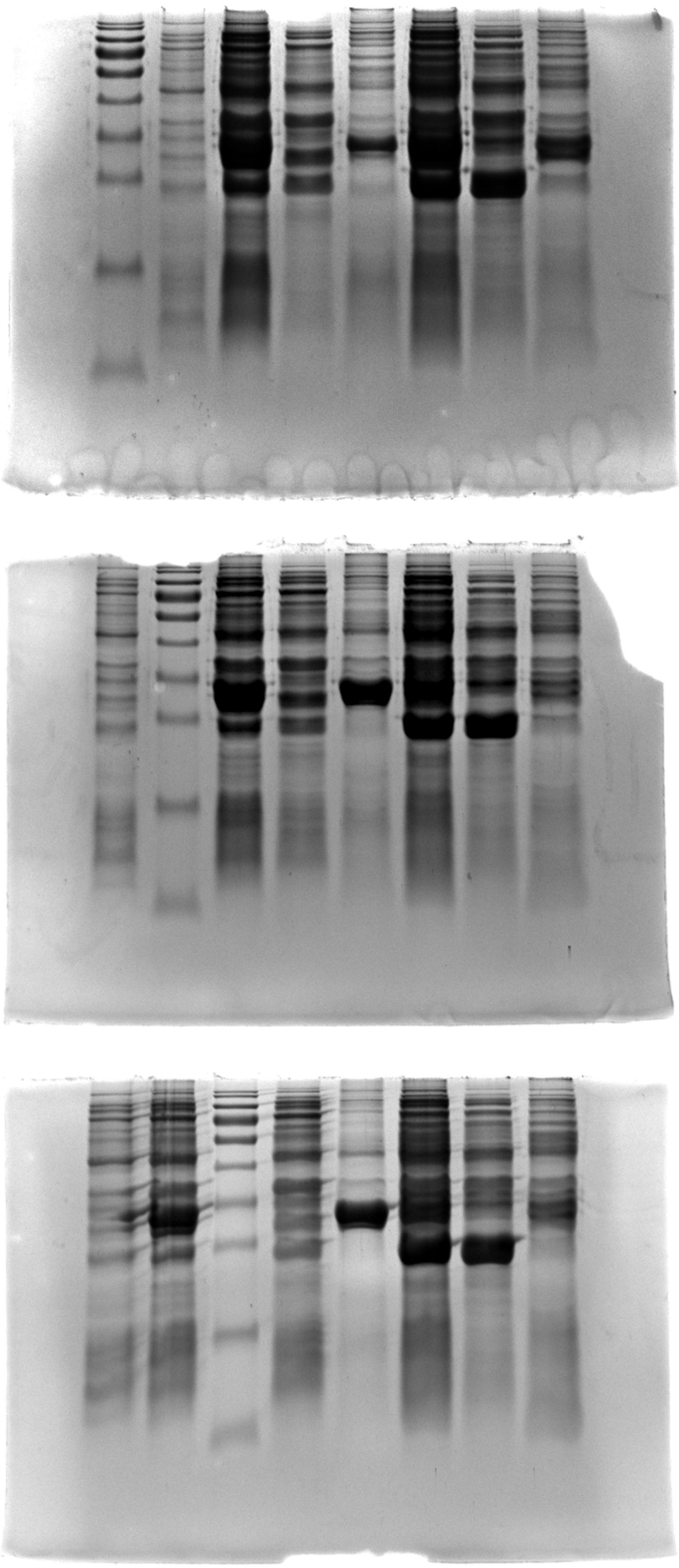

Fig. S2 c

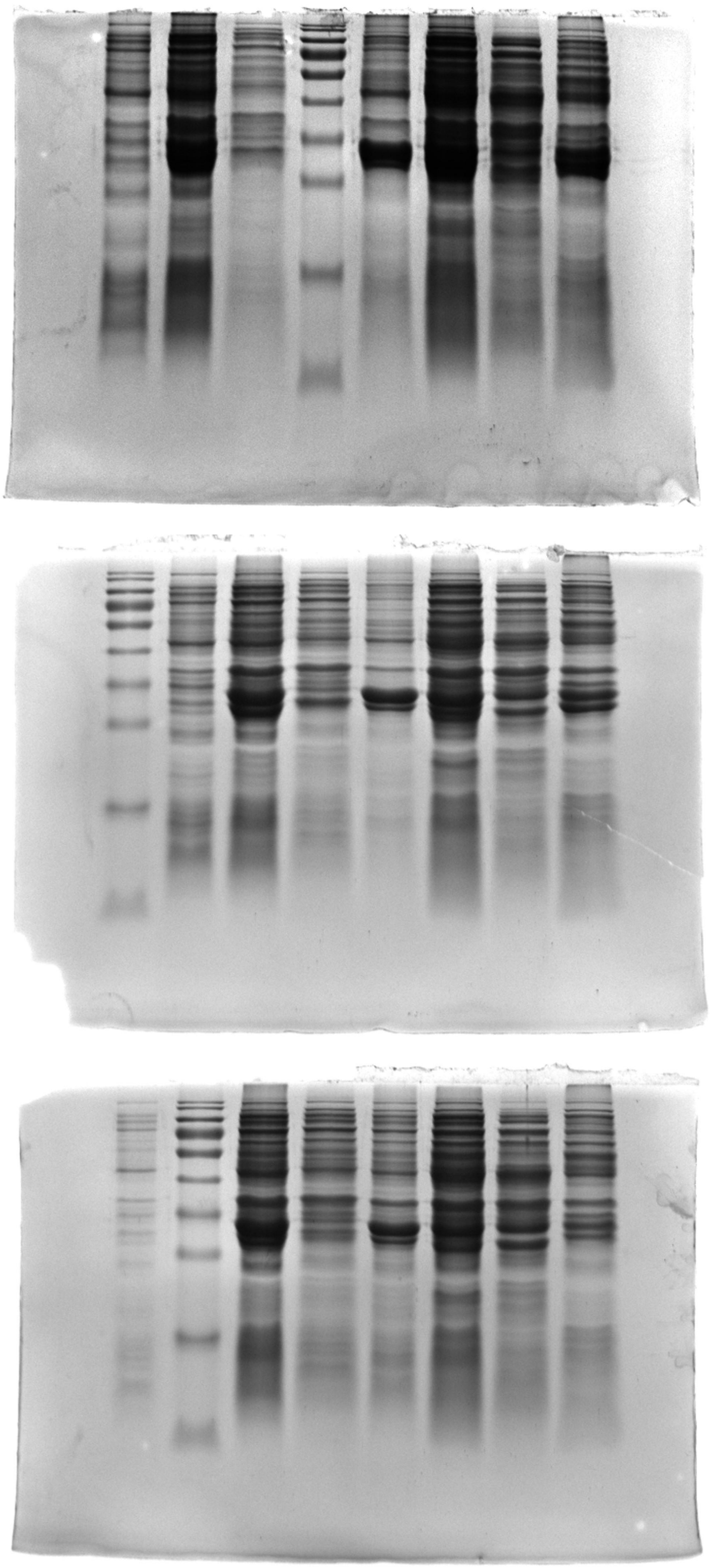

Original uncropped photos of western blots made on the iBright 750 (Thermo Scientific)

Chemiluminescence

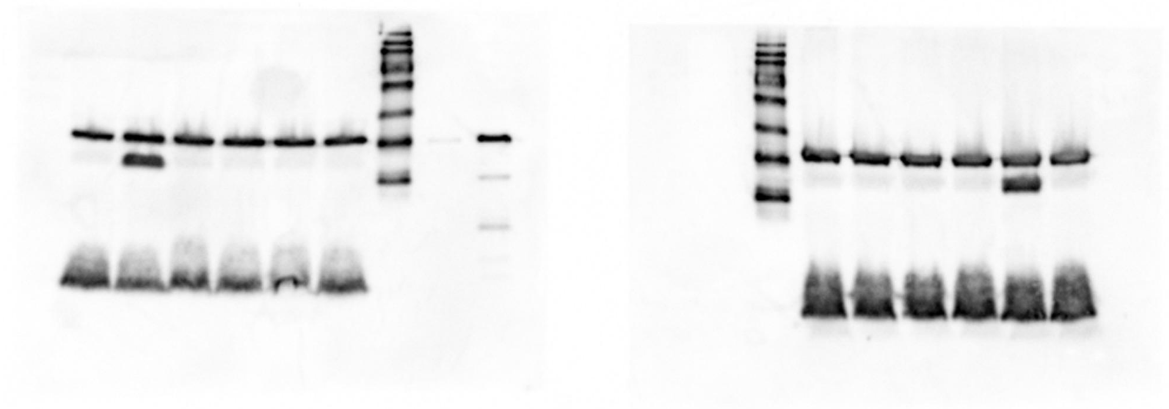

Overlay with the membranes.

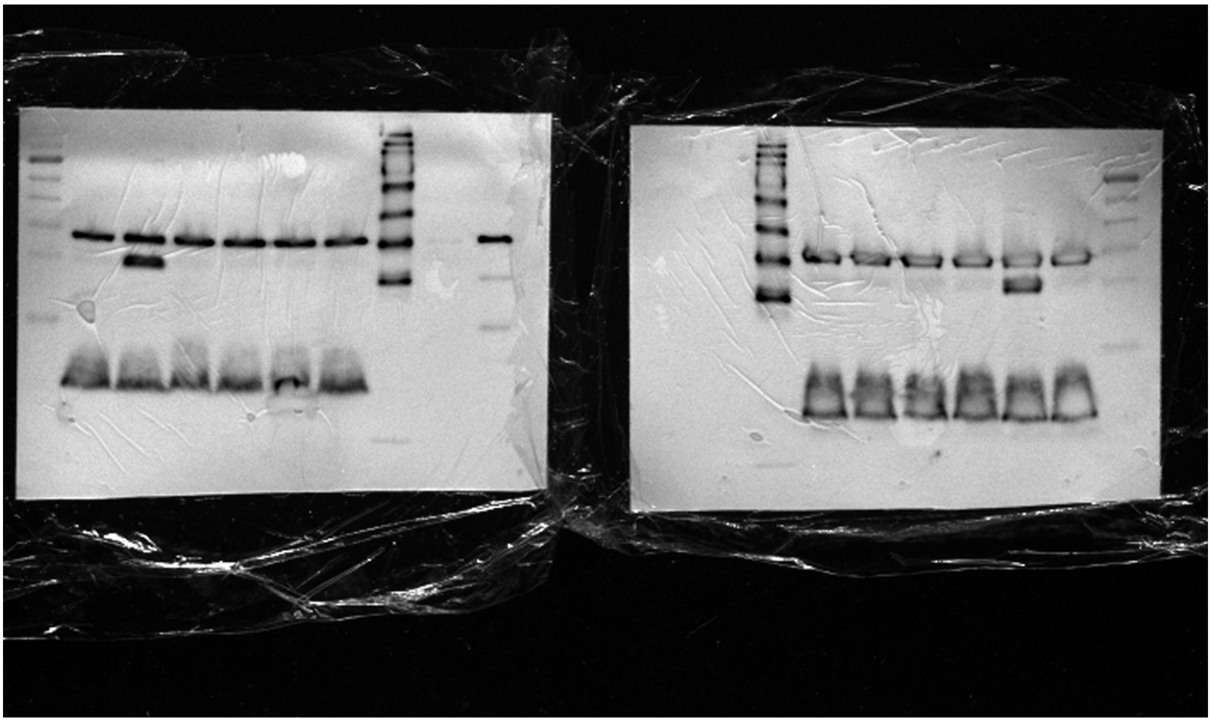

Supplement: Supplementary file 2 — Supplementary Information 2. [file 41598_2024_58492_MOESM2_ESM.pdf]
